# Aggregated N-of-1 trials for unlicensed medicines for small populations: an assessment of a trial with ephedrine for myasthenia gravis

**DOI:** 10.1186/s13023-017-0636-y

**Published:** 2017-05-12

**Authors:** Stephanie S. Weinreich, Charlotte Vrinten, Marja R. Kuijpers, Alexander F. Lipka, Kirsten J. M. Schimmel, Erik W. van Zwet, Christine Gispen-de Wied, Yechiel A. Hekster, Jan J. G. M. Verschuuren, Martina C. Cornel

**Affiliations:** 1 Department of Clinical Genetics, Amsterdam Public Health research institute, VU University Medical Center, Amsterdam, The Netherlands; 2 Department of Care, National Health Care Institute, Diemen, The Netherlands; 3 Department of Epidemiology and Public Health, University College London, London, UK; 4 Department of Neurology, Leiden University Medical Center, Leiden, The Netherlands; 5 Department of Clinical Pharmacy and Toxicology, Leiden University Medical Center, Leiden, The Netherlands; 6 Department of Medical Statistics and Bioinformatics, Leiden University Medical Center, Leiden, The Netherlands; 7 Medicines Evaluation Board, Utrecht, The Netherlands

**Keywords:** N-of-1 trials, Health insurance reimbursement, Drug approval, Ephedrine, Myasthenia gravis, Off-label use, Drug rediscovery, Personalized medicine, Technology assessment (biomedical), Rare diseases

## Abstract

**Background:**

Inexpensive medicines with a long history of use may currently be prescribed off-label for rare indications. Reimbursement is at the discretion of health insurance companies, and may be unpredictable*.* The example addressed was ephedrine as add-on treatment for myasthenia gravis. Stakeholders from academia, a patient organization, the Dutch National Health Care Institute (NHCI) and Dutch Medicines Evaluation Board (MEB) advised on the trial design. The NHCI and MEB agreed to provide scientific advice on the suitability of the evidence generated by the trial, for regulatory decisions. This paper describes the feasibility of the trial and the utility of its aggregated results.

**Results:**

The trialists experienced the trial as feasible. Retrospective interviews showed that the trial as performed was acceptable to patients. The treatment effect in the primary outcome measure, muscle strength, was statistically significant when inferred to the population level, though the effect size was modest. Secondary outcomes were statistically significant in a preplanned, fixed effects analysis within the four patients. The NHCI advised that it could potentially make reimbursement decisions based on the Fitting Evidence framework, should the trialists decide to apply for reimbursement. The MEB advised that for a licensing decision, the N-of-1 design is a last-resort option for demonstrating treatment benefit in a rare disease. N-of-1 trials alone do not provide enough evidence on potential risk. The MEB found the current trial inconclusive. It suggested doing a 2-armed trial of longer duration, possibly with a different outcome measure (postponement of corticosteroid use). It suggested engaging a consultancy or commercial sponsor, should the trialists decide to seek market authorization of the drug.

**Conclusions:**

In theory, evidence from aggregated N-of-1 trials is suitable for use in licensing and reimbursement decisions. The current example illustrates differences in interpretation of N-of-1 results by health authorities. In the era of personalized medicine, consensus is required on the interpretation of data from study designs geared to small groups. Demonstrating effectiveness of inexpensive medicines in small populations may require involvement of non-commercial parties, to preserve affordability.

**Electronic supplementary material:**

The online version of this article (doi:10.1186/s13023-017-0636-y) contains supplementary material, which is available to authorized users.

## Background

Current treatment paradigms for rare diseases include not only expensive, novel orphan drugs but also cheaper medicines with a longer history of use. The latter are often prescribed off-label for rare indications, whereas they may be licensed for other, more common indications. Some medicines prescribed for small patient groups are not licensed at all; they are compounds prepared by pharmacies. An example is ephedrine for myasthenia gravis. Medical scientific societies for rare diseases include off-label and compounded medicines in treatment guidelines, underpinned with the evidence available. Nevertheless, reimbursement can be a problem. Current policy in the Netherlands discourages reimbursement of off-label and compounded medicines in favor of licenced ones because the former may have not been systematically assessed for effectiveness [1, 2]. Other EU countries have similar policies [3, 4]. The next paragraph details specifics of the Dutch health care system which hinder the reimbursement of moderately priced, older medicines for rare diseases. Then it is explained how this research contributes to a roadmap which can be used in other countries as well, suggesting opportunities for “rediscovery” of cheap old drugs for rare disease indications. To help generalize from the situation in the Netherlands, key concepts are shown in italics.

Every Dutch resident is obliged to take out *basic health care insurance*. It is sold by private companies and it covers a uniform package of health care, the scope of which is explicated by the National Health Care Institute (NHCI, Zorginstituut Nederland). As far as outpatient medicines are concerned, reimbursement of off-label, unlicensed or compounded medicines is not defined by the package but is *at the discretion of the health insurance company*, on a case by case basis. Reimbursement of medicines taken in hospital are beyond the scope of this paper. Statistics on reimbursement decisions for such treatments are not in the public domain, but there is evidence of variability: concerning compounded medicines, the government recently expressed the need for insurance companies to harmonise their reimbursement policies [5]. Furthermore, it is the experience of the authors that when specialized physicians in tertiary treatment centers write a reasoned request for reimbursement of an off-label, unlicensed or compounded product on behalf of a patient with a rare disease, *the insurance company’s decision is unpredictable*. Occasionally the NHCI issues guidance on whether an off-label indication or a compounded medicine can be deemed rational pharmacotherapy^1^ and therefore reimbursable. For a positive decision, published evidence of effectiveness must be available. However this procedure is reserved for indications with a prevalence below 1:150,000 or expensive medicines (e.g., biologicals licensed for prevalent inflammatory diseases). This leaves a *regulatory gap for many rare diseases* (defined in European law as less prevalent than 1:2000) [6] regarding, notably, *moderately priced treatments*. One solution might be to get such products on license. Indeed, drug licensing authorities have started to encourage licensing of older compounds or products, often termed drug rediscovery or treatment repositioning [7]. Naturally, this pathway requires demonstration of efficacy. Compiling evidence, even when it is old evidence, is labor intensive. Ironically, putting previously off-label and compounded products through the licencing procedure has raised their price, sometimes to the point that they are so cost-inefficient that they are not reimbursed [8].

Any stakeholder who wants to create evidence on the effectiveness of a medicine for a small group, will strive for trial efficiency. When only a small sample of patients can feasibly be recruited, there is the risk that even if the treatment actually is effective, the evidence from the trial may not meet statistical convention (the point estimate of the effect may be clinically relevant but p will be larger than for example 0.05), in other words, the trial may be underpowered. Among the trial designs which are suitable for small groups is the N-of-1 design [9–13], and this has been recognized at the regulatory level [14, 15]. An N-of-1 trial is a randomised, controlled, multiple cross-over trial in a single patient. The treatment of interest and control (e.g., placebo) are randomised over multiple treatment periods. The more cross-over cycles, the more precise the effect estimate. N-of-1 trials are suitable to test treatments with quick onset and a short half-life for chronic, stable conditions [16]. When these conditions apply, N-of-1 trials provide the highest form of evidence for individual patients [17, 18]. When the same N-of-1 trial protocol is used for several individuals, aggregated data can be used to estimate the treatment effect at population level, as robustly as with traditional parallel-armed RCTs [19]. Moreover, because each patient provides several sets of matched data to each trial “arm”, generally smaller sample sizes are needed than in conventional RCTs [16, 19, 20]. However, compared to equally powered trial designs addressing the same clinical question, N-of-1 trials may be more burdensome for patients if the trial lasts longer (due to multiple cross-overs) and requires intensive data collection (e.g., diaries, outpatient visits) [16, 21]. A systematic review illustrates that N-of-1 trials have been published for many indications [22], but there are almost no published examples that evidence from N-of-1 trials was used for a decision for licensing or reimbursement of a treatment for a small patient group. The single example, to our knowledge, is that the licence of immunoglobulin was extended to chronic idiopathic demyelinating polyradiculoneuropathy on the basis of an industry-sponsored N-of-1 trial [23]. It has been proposed that N-of-1 trials be used more often for “treatment repositioning”, a goal which is echoed in this study [10, 17].

A preliminary scoping of regulatory and medical stakeholders in the Netherlands revealed unfamiliarity with the statistical aspects of N-of-1 trials but also consensus that the suitability of N-of-1 trials for regulatory decisions should be explored in practice, in the context of emerging regulatory policy. (Vrinten, in preparation). Evidence from a series of N-of-1 trials has so far not been assessed under the NHCI’s “Feasible Information Trajectory”, an algorithm to determine which type of evidence is feasible for a particular indication [24]. It has also not been examined from the perspective of the Dutch Medicine Evaluation Board’s (MEB’s) policy to foster drug rediscovery [7, 24]. (The MEB regulates the market approval of medicines.) The scoping also identified suitable indications for N-of-1 trials, including symptomatic treatment of myasthenia gravis. Myasthenia gravis is a rare neuromuscular disease which is primarily treated with acetylcholinesterase inhibitors. In case of inadequate response, corticosteroids or other immunosuppressive medication are used, but their side effects may be serious [25]. Ephedrine is mentioned in an international guideline published in 2010 [26]. There is anecdotal evidence that it can reduce, postpone or prevent the need for immunosuppressive therapy when added to acetylcholinesterase inhibitors or low-dose prednisone but a Cochrane review found no evidence on ephedrine from randomized clinical trials [27]. No oral ephedrine preparation is currently licensed for humans in the European Union for myasthenia gravis [28] and pharmacies in the Netherlands must either import tablets from Spain (50 mg tablets, Laboratorios ERN, licensed in Spain for the treatment of asthma and hay fever) or compound ephedrine capsules themselves. The reimbursement of ephedrine tablets for myasthenia gravis is not guaranteed in the Netherlands. Taken together, ephedrine for myasthenia gravis emerged as a relevant and suitable case, and we designed a protocol for a series of N-of-1 trials [25]. The clinical results have been reported elsewhere [29]. The aims of this study are: (1) to evaluate the feasibility of an N-of-1 trial protocol for determining efficacy of ephedrine as an add-on treatment in myasthenia gravis, from the trialists’ and patients’ perspective, (2) to examine whether a series of N-of-1 trials provides suitable and sufficient evidence for the NHCI to make a decision on reimbursement of the unlicensed product, and (3) to explore the suitability and sufficiency of the evidence for market authorization of ephedrine for myasthenia gravis under current regulatory pathways, based on advice from the MEB.

## Methods

### Stakeholder involvement in study design and preparation

In order to enhance the trial’s acceptability to patients and to generate data relevant at the regulatory level, the clinical trial protocol was developed by a multi-stakeholder team including clinical and other academics, experts affiliated with NHCI and the Dutch patient organisation for neuromuscular disorders (Spierziekten Nederland). The MEB advised on the regulatory suitability of the methodology as presented in a draft of the protocol. Next, educational workshops were organized at NHCI and MEB before the trial data were available, in order to raise awareness of the statistical aspects of N-of-1 trials. The workshops explained how aggregated N-of-1 trials can be analysed at the level of the trial patients and at the population level. Briefly, in the first case a linear model is fitted with fixed effects for treatment and patient. In case an effect is found in the trial patients, the effect in the population can be inferred by fitting a linear mixed model with fixed effects for treatment and patient and a random treatment-by-patient interaction. Finally, the research team liaised with NHCI and MEB to create a procedural format, i.e., apply for scientific advice, for assessing the utility of the trial results in relation to reimbursement and licensing. It was agreed to make flexible use of two existing procedures: tailored scientific advice from MEB to academic researchers and joint scientific advice from MEB and NHCI.

### Feasibility of the trial protocol

The outcome measures for feasibility were prespecified in the published version of trial protocol [25] and/or in the protocol overview registered through EuDRACT [30].

### Trialists’ perspective

The protocol prespecified that the experiences of the physician and pharmacist would be evaluated. In the course of the study, it emerged that it would be useful to also include the experiences of the trial statistician and project coordinator. The quantitative measure (prespecified) was time spent on the trial. Qualitative exploration of trialists’ experiences was prespecified to be based on experiences reported during monthly project meetings, according to the protocol. In practice, the meeting minutes lacked richness and reflexivity. Therefore at the end of the project each trialist (JV, SL, KS, EvZ) and the project coordinator (SW) wrote a concise description of experienced obstacles, solutions found and suggestions for future N-of-1 trials. One author (SW) compiled these experiences after member checking with the trialists.

### Patients’ perspective

Quantitative measures were the numbers of eligible patients, patients recruited and patients who completed their n-of-1 trial; the total number of completed cycles (a cycle consisting of 5 days of active treatment or placebo, 2 days washout, and 5 days of the opposite treatment plus 2 day washout), and the number of patients who decided to participate in the open-label extension phase. In addition, a prespecified threshold of feasibility was that on average each patient had to complete at least two randomized treatment cycles [30]. All measures were analysed descriptively.

After the trial, patients’ experiences were explored qualitatively by individual patient interview and are reported in accordance with COREQ standards [31]. Patients who had completed at least one treatment cycle were eligible. Informed consent for the interview was integrated in the informed consent for the clinical trial. The interviews were conducted 1 to 2 weeks after the outpatient visit when patients learned their unblinded trial results and decided whether to participate in the open-label extension. Patients were not acquainted with the interviewer (SW). The interviews were semi-structured, based on an interview guide with questions and prompts. The topics were based on theoretical benefits and burdens of N-of-1 trials [16], published studies on patients ‘and carers’ experiences with N-of-1 trials [21, 32], and process-related aspects of the trial at hand. The interviews were held by telephone, audio recorded (after verbal affirmation of consent) and lasted up to 30 min. Audio recordings were transcribed by the interviewer and copied into analysis software (Atlas.ti. 7 for Windows, Atlas.ti Scientific Software Development GmbH, Berlin, Germany). One author coded the data (SW) and together with a second author (CV) generated a list of themes. Where illustrative quotations are presented, the patients are identified by a number unrelated to the numbering in the publication on the clinical results. Protecting patient confidentiality is a particular concern in reporting N-of-1 trials, where numbers of patients tend to be small while data collection is intensive [17].

### Utility of the trial for reimbursement and licensing

The regulatory questions prompting the study were addressed by eliciting joint scientific advice from MEB and NHCI. The MEB charges graduated fees depending on the scope of the questions and the type of applicant. The advice for ephedrine for myasthenia gravis was “customized scientific advice”, a reduced fee category (€1750) introduced in 2015 to encourage innovation e.g., in drug rediscovery.

First, the applicants submitted a briefing document which presented the evidence of beneficial effects and risks of the treatment, based on published scientific literature and the N-of-1 trial results (the briefing document is shown as Additional file 1). The agencies were requested to answer the following questions (Table 1).

**Table 1:** Questions submitted to the reimbursement and licensing authorities for scientific advice

National Health Care Institute	Medicines Evaluation Board
Could the aggregated data from the series of N-of-1 trials, as presented in the briefing document, play a role within the framework of a reimbursement advice, in case a medical professional society requests such an advice at a later date?	Are the data in the briefing document sufficient for a benefit/risk evaluation (for licencing) and if not, what else is required?
If so, in what way could it play a role and what might the reimbursement advice be?	
If not, why not? What level of precision in the data is needed for a reimbursement advice at the population level?	In case the level of precision in the data is insufficient for a benefit/risk evaluation (for licensing), how many more patients would need to be included in the aggregated N-of-1 trial to enable MEB to make a judgement? What specific outcome or type of analysis would be recommended as the basis of a new sample size calculation?
	What would be the recommended regulatory route to get this indication on-label? Which would be the preferred route: ephedrine tablets imported from Spain or a possible future product to be compounded by a Dutch GMP-certified pharmacy (LUMC)?

After receiving the briefing document, the MEB further requested anonymized individual patient data, to help assess whether the conditions for N-of-1 trials had been met. These data were submitted. Next, the applicant was invited for a formal discussion meeting. Finally, each agency issued its own written response. One author (SW) extracted themes from the responses and together with a second author (MC) compared the responses of each agency. The results were member-checked with co-authors from each agency.

## Results

### Brief overview of clinical results

The clinical results of the trial have been reported elsewhere [29]. Briefly, four patients completed three treatment cycles each containing a treatment period with ephedrine and placebo in addition to their usual medications. A modest but statistically significant effect of ephedrine was demonstrated on the primary outcome of muscle strength within the four trial patients and also when inferred to population level. Secondary outcome measures were statistically significant in the trial patients but not after extrapolation to population level. Side effects were mild. Based on their trial outcomes, three of the four patients decided to continue with ephedrine treatment in the open-label extension phase of the study. The fourth patient declined to due to multiple side effects, which were individually mild, but together outweighed the experienced benefit of treatment.

### Feasibility

#### Trialists’perspective

The project encompassing the trial and its evaluation was planned to take 12 months but took 18 months. The institutional review board (IRB) approved the trial 8 months after the project’s start. About 80% of the project grant (€50,000, publicly funded) contributed to salary costs of a neurology resident and the project coordinator/interviewer. Table 2 shows the total number of hours which the most intensively involved members of the team devoted to the project during its 18-month run. In addition, other team members contributed substantial time in kind (see the Authors‘Contributions and Acknowledgements sections). Moreover, the trial would not have been possible without a previous, publicly funded project which demonstrated regulatory interest in N-of-1 trials (Vrinten, in preparation), produced a systematic review of ephedrine for myasthenia gravis [27] and laid the groundwork for the trial protocol [25].

**Table 2:** Hours spent on the N-of-1 trial by the most intensively involved team members^a^

Neurology resident (main trial physician)	Project coordinator/interviewer	Senior neurologist	Statistician	Pharmacist
1131	328	101	75	41

^a^Hours include clinical, research and administrative tasks. Source: report to funding organisation ZonMW

The clinical researcher, a neurology resident experienced in neuromuscular disease, spent more time on the trial than anticipated. Because myasthenia gravis is rare to begin with, it was challenging to find patients who met the inclusion criteria (relatively stable disease, insufficient response to standard medication, no contra-indications for ephedrine). Also, the procedures for IRB approval and trial registration in (international) registries were as time consuming as for a trial with more participants. On the other hand, due to the small number of participating patients and the short duration, the trial could be executed by a small team and was relatively easy to conduct.

The project coordinator spent more time on the project than anticipated due to the complexity of liaising with NHCI and MEB to find a procedural format for assessing the utility of the trial results. Also, there were 6 months’ extra of management tasks.

For the hospital pharmacist the trial took more time than expected due to the poor availability of ephedrine tablets and searching for alternatives. In the end ephedrine tablets were imported from Spain and repackaged during the trial. Positive factors for the time demands put on the pharmacist were the small number of trial patients, the prompt arrival of the imported tablets and the limited compounding tasks required.

The statistician performed the usual tasks of sample size calculation and developing and executing an analysis plan. In addition he supervised a Master student who analysed a set of aggregated N-of-1 data, unrelated to the trial at hand, to provide illustrative material for the preparatory workshops for the regulatory agencies (see Methods).

#### Patients’ perspective

All four patients completed all three treatment cycles, thereby meeting the prespecified, quantitative feasibility criterion. The following sections present results from the patient interviews. Eight themes relate to the trial’s feasibility and one to its utility. Of the nine themes, the last three are specific to the blinded, multiple cross-over trial design. Because of their special relevance, they are supported with illustrative quotes.

##### Information about the trial

Patients thought that the written and oral information provided before the trial was clear, and that the trial had proceeded accordingly. One patient particularly appreciated the time taken by the neurology resident to explain everything beforehand.

##### Safety concerns

Some patients had safety concerns before the trial. One patient noted that side effects would only be measured on 2 days, referring to the clinical measurements taken on the first day of each week in the first treatment cycle. However she did not worry much about side effects, finding it reassuring that ephedrine has a fast offset. Another patient was worried about interaction between study medication and drugs she was already taking. It was reassuring to her that she could discuss these concerns with the trial physician. A third patient’s main concern was whether physicians could be reached during the trial. On the basis of the information she had, she thought the physicians’ availability was well organized.

During the trial patients had varied experiences regarding the availability of physicians. The patient who had been concerned beforehand expressed appreciation for the fact that she could almost always reach physicians during the trial. However another patient felt that it had taken a long time to get in contact with the trial physician, at a moment when she was concerned about side effects of trial medication. At that juncture she even considered withdrawing from the trial.

##### Time investment and trial organisation

Before the trial, patients viewed the time to be invested as considerable, but several factors made it acceptable: the fact that travel expenses would be reimbursed, that hospital visits could be planned on a day when the participant didn’t work, and, in one case, that time spent on the trial counted as part of a work rehabilitation program. After the trial, a participant suggested that the weekly measurements be done in each patient’s town of residence, provided the measurements would be done by the same (group) of professionals; after all, the trial concerns scientific research.

Participants were positive about the days when they were hospitalized at the beginning of each of the first two treatment weeks. They described the days as thoughtfully arranged, fine, and pleasant. One participant noticed that a few hospital workers, such as the nurses who took the measurements, were not aware of the trial and expressed curiosity about it. The participant wasn’t bothered by this but she suggested informing hospital workers in advance.

Two participants went through the trial at the same time and thus came in contact with each other at multiple outpatient visits. It was agreeable to be able to share experiences and it was reassuring when they matched. However one participant felt confronted that the treatment seemed very effective for the other patient but not herself. She suggested that doctors estimate in advance whether individual patients can handle such a disappointment, before scheduling adjacent appointments for multiple trial patients.

##### Breaks during the trial

In one patient’s case the trial was interrupted because an outpatient visit would fall on a holiday. This patient felt that her overall trial results would have been clearer if the cycles had been uninterrupted, because the disease fluctuates. Another participant paused the trial due to family circumstances. She experienced the events in the family as difficult but she was motivated to start the trial again because of the trial’s scientific purpose, among other reasons.

##### Ease of use of trial medication

One participant spontaneously stated that she did not find it easy to swallow the trial capsules (containing 25 mg ephedrine or placebo); she found them very big. She then put this into perspective by saying that she even splits paracetamol tablets.

##### Side-effects questionnaire

Participants filled out a questionnaire about side effects three times a week. They thought the questionnaire was clear. A patient who did not experience side effects finished the questionnaire quickly, in a minute, while a patient who had many side effects found it rather a lot of work to fill in the questionnaire. Nevertheless she suggested that the questionnaire also be filled in on weekends (the washout period) because she experienced effects of trial medication then too. Another trial participant incidentally also mentioned that she noticed effects of trial medication during the washout period.

One participant liked it that there was room for extra elucidation in the questionnaire. She believes that experiencing less or more energy is very important to patients with myasthenia gravis, although she noted that this is subjective and therefore probably cannot be scientifically underpinned. She thought it worth considering whether to add a question about experienced energy at the end of the questionnaire.

##### Guessing trial medication

During the outpatient visit at the end of each treatment period, participants were asked whether they thought they had received ephedrine or placebo. Most participants were positive about this task and were not afraid they might give the wrong answer. One participant liked the task because she could answer on the basis of the energy she had experienced, an outcome which was very important to her but which had not been asked about anywhere else in the study. Some participants indicated that their experience of guessing changed in the course of the trial. One participant described how she found it suspenseful to sense the test medication, at the beginning of the trial:

*“Suspenseful. In the sense that, you don’t know whether you’re taking the drug [ephedrine]. I had placebo the first day. But you’re constantly watchful. Do I notice anything? Am I feeling anything? Is this it, then? So, yes, it’s suspenseful. Fun too, but also suspenseful.” (Patient 4)*

Later, guessing got easier for this participant, because she always experienced side effects of ephedrine within half an hour. For another participant, however, guessing became more difficult in the course of the trial. She ascribed this to the difference in circumstances between the days of planned hospitalization and daily life.

*“In the hospital I knew right away…, but okay, then you’re focused on, what are you going to feel? What are you going to notice? You don’t really have anything to do there, because you’re just sitting there in a room. So, you’re thinking like, so what’s actually going to happen? Yeah, and then you go up some stairs, and you think wow! [laughs] That’s a lot easier than usual! At home you’re more in your daily routine, there are two kids running around, so you don’t really have time to stop and consider, what is it this time? And of course you’re moving around more, too. Sometimes I was like, well this is it [ephedrine], and sometimes I thought, well, I don’t know. And I actually I found that a little more frustrating.” (Patient 1)*

The participant explained further that she didn’t feel the frustration during daily life, but rather when she had to state her guess to the physician.

##### Learning the results and therapeutic decision

Two weeks after finishing the blinded treatment cycles, participants had a meeting with the trial physician. The physician showed the patient the unblinded, individual trial results and presented the possibility of continuing with ephedrine treatment during a 6-month open label extension study. The interviewer later asked patients which emotions they felt before the meeting. These feelings might be related to their treatment preference.

Two participants described feeling primarily curious about the trial results. One of them didn’t know whether she wanted to continue with ephedrine, while the other was convinced that she wanted to continue with ephedrine treatment.

Another participant, who was strongly convinced of her treatment preference, felt primarily impatient for the meeting to take place.

*“I usually… went every week, but the week of the meeting I couldn’t go, so it was postponed by a week. So then my symptoms came back again, in abundance. I was very glad I could go there and that I got the tablets [right away]. So, no, I wasn’t nervous. I felt like, come on, give me those pills!”(Patient 2)*

Another participant primarily felt the burden of a difficult decision. She did not have a clear treatment preference because she felt that she had experienced both effectiveness and side effects of ephedrine.

Participants described that the physician provided information about their treatment options along with arguments they could use to support their decision. All participants experienced the final decision as their own choice. One participant had mixed feelings about this; she would have found it easier if the physician had been more directive. Another patient, who had doubts about continuing with ephedrine, made the final decision after meeting with another physician.

##### The trial’s utility

Patients had expected various types of advantages from trial participation. Some were personal: the chance to reduce their symptoms, avoid future use of prednisone or taper current prednisone use. Also, some patients spontaneously mentioned altruistic motives. Some wanted to help science or other patients. One patient described a sense of duty to participate because of the rarity of the disease. Another felt positive about the trial because she had always enjoyed participating in medical education, e.g., clinical lectures. Moreover she saw her participation as a way of expressing her gratitude for the good care she had received at the hospitals which were collaborating in the trial.

In retrospect, all patients felt that the trial had been worthwhile. For some, the personal therapeutic value was clear:

*“For sure. I didn’t go through it for nothing. It went better than I expected. And well, now I have the pills. And that, yes, it [ephedrine] works well. I’m not completely symptom-free, but for the most part I am.” (Patient 2)*

*“… because I do think that the medicine is something that… that can at least provide relief.” (patient 3)*

One patient underpinned the therapeutic advantage with the trial’s objective outcome measures:

*“That it helped me after all. Because otherwise you wouldn’t have seen it in my results.” (Patient 1)*

Patients also felt that their participation was valuable for others. One spontaneously mentioned the importance of the study for the availability and reimbursement of ephedrine. Finally, one patient put her own disappointing trial results into perspective by considering their utility for science:

*“It’s better when it’s effective and it has few side-effects. So there were times when I thought, well, what’s the value of my participation? But afterwards I think it was valuable because this way they can also chart, [that] for some patients it works like this, for others like that. So I think it was valuable after all.” (Patient 4)*

### Regulatory utility

The regulatory agencies NHCI and MEB provided scientific advice on the evidence for reimbursing and licensing ephedrine as add-on treatment for MG. The NHCI formulated its advice according to its framework of “Established medical science and medical practice” [33]. The MEB’s advice did not refer to a framework for acceptability of evidence, but did refer to the European legal basis for various routes to licensing. Table 3 thematically summarizes the scientific advice given by NHCI and MEB, including methodological points from the MEB’s advice during the design stage of the trial. The following sections compare the positions of the two agencies by theme.

**Table 3:** Regulatory perspectives on the utility of the N-of-1 trial data

Themes and subthemes	Response of NHCI regarding reimbursement	Response of MEB regarding licensing
Considerations for accepting evidence from N-of-1 trials^a^		
Condition and treatment	Chronic, stable disease; fast on- and offset of treatment	Chronic, stable disease; fast on- and offset of treatment effect
Prevalence	A traditional RCT is not feasible due to small patient numbers. The Feasible Information Trajectory [24] indicates that the N-of-1 design is acceptable.	General approaches to rare conditions exist. ^b^ The N-of-1 design is a last resort option for rare conditions
Generalizability to population level	The results of individual patients’ trials must be aggregated to allow a statement about effectiveness at the population level.	Patients should be sufficiently diverse (e.g., characteristics, context of care).^c^ Individual results should be shown.^d^ An overall mean may not be interpretable if there is high heterogeneity.^e^
	-	The number of patients involved [is] crucial for generalization of effect
	Consider how many measurements are needed per patient and the total number of patients to be included.	The number of periods assessed must be sufficient
	-	Multiple study sites are preferable
Other	The evidence of effectiveness must be published in a peer-reviewed journal.	N-of-1 trials are not suitable for substantiating safety in a benefit/risk assessment for market authorization. Safety needs to be substantiated otherwise.
Evidence from this series of trials with ephedrine as add-on for myasthenia gravis		
Patient characteristics and external validity	Patients were receiving usual care as recommended in international guidelines, thus the usual care is acceptable. One patient was taking low-dose prednisone and an immunosuppressant during the trial. How might this have influenced the results? All patients were female. Are the results generalizable to males?	The MG population in this study is heterogeneous with differences in disease duration, baseline scores, concomitant treatment. Hence there should be a justification to which extent these results based on 4 subjects can be extrapolated to the general MG population.
Comparator	Placebo control is suitable for answering the question whether adding ephedrine to usual care is more effective than not adding it.	-
Outcome measures	QMG is acceptable as primary outcome measure because it is endorsed by MGFA. Clarification is desirable on QMG’s validation and correlation to EQ-5D, in case EQ-5D cannot be used for this indication.	-
Timing	Is a 5-day treatment period sufficient to show a clinically relevant effect on the chosen outcome measures, considering that previous studies in myasthenia gravis used 14-day treatment periods?	-
Variability of observed effects within patients: questioning the validity of the N-of-1 design for this indication		On a group level, ephedrine tended to be effective based on the effect estimators, but responses within patients were highly variable. In Figure one of the Briefing document [Additional file 1] the mean of the placebo episodes and the mean of the ephedrine episodes per patient are presented. However, the observed scores within these episodes were rather variable. A clear, consistent treatment effect within a patient was not observed. In response the Applicant stated that motor performance in MG is highly variable over the day. This challenges whether the assumptions of the N-of-1 trial can be met as patients appear not to return to baseline levels in motor performance after stopping treatment. In theory, MG could have met the assumptions for an N-of-1 trial, but this was not clearly observed in the data. Shortly, individual patients could not be classified as responders. Moreover, the severity of motor symptoms in MG may vary over the days. It may even vary within a day, as it will decrease during the day especially in case of exercise. As such, it may be questioned whether patients included had the correct baseline characteristics: was disease activity constant? Within each patient, responses for the different scales were not consistent, adding to the variability of the data, hampering the interpretation of the results.
Clinical relevance and statistical significance of effect in primary outcome measure	The clinical relevance of the found effect, 1 point reduction in QMG, requires further support. For the initial treatment of myasthenia gravis, a reduction of 3.5 points on the QMG scale is clinically relevant. Is a reduction of 1 a small, average or large effect? Expression as a SMD would be helpful. Is there literature to support its relevance for add-on ephedrine? Is a MCID known for QMG?	Although significant p values were presented for the treatment effect of add-on ephedrine compared to add-on placebo, the clinical relevance of the effects was considered inconclusive. Effect size is small which questions the clinical relevance of effects. The initial, postulated minimal clinically important difference by the Applicant was 3.5 points on the QMG scale. This was based on literature and was not met. In the discussion meeting the Applicant stated that this might have been too ambitious and a lower effect size may still be relevant. However, this prompts a justification why the observed differences would be clinically relevant.
Interpretation of statistical results in the trial patients compared to inference at population level	-	-
Outcomes not addressed in the trial	The trial at hand is not suitable for answering the question whether ephedrine postpones or prevents the use of treatments with a higher risk profile. NHCI wondered whether applicants could provide insight from this series of trials or literature.	The argument that the treatment might reduce or postpone corticosteroid use should be demonstrated.
		Clinical relevance should also be discussed in terms of benefit/risk. (N-of-1 trial not suitable, see above). Safety needs to be substantiated otherwise. The effect size should be weighed against safety e.g., long-term cardiovascular risks. Applicant did state that intermittent use was aimed for.
Sufficiency of the evidence for a decision	NHCI cannot at present give a definitive answer whether this series of N-of-1 trials shows that the treatment in question is “Established medical science and medical practice” [33]	The data from the trial are not adequate to base a marketing authorization upon.
Desired level of precision/how many patients still to include (for MEB only): What outcome or type of analysis would be recommended?	It is the applicants’ responsibility to demonstrate that the statistical methodology as applied to this aggregated N-of-1 trial design, is the correct methodology to enable a reliable statement about an effect at the population level.	The observed data do not allow the conclusion that the conditions for a N-of-1 trial have been met. Firm recommendations on what would suffice [how many more patients to include in the aggregated N-of-1 trial and/or what specific outcome or type of analysis] cannot be made as it depends on the reasons for failure of the current study design [to show clinical relevance] [e.g., insufficiencies in the trial design, inclusion of an incorrect patient population and/or in fact the drug’s being ineffective]. Options might be inclusion of a positive control with a clear symptomatic effect (e.g., N-is-1 trial with placebo/ephedrine/acetylcholine crossovers) in combination with selection of a more responsive patient population. Revision of the inclusion criteria of the study population to assure a constant disease activity also may increase the probability of showing a symptomatic treatment effect. A different study design e.g., a parallel group trial with a longer treatment duration where the day to day variability in scores can be averaged, also may be considered.
	At the present stage it could not make a decision under the framework of “Established medical science and medical practice” on whether ephedrine is reimbursable for the indication under consideration, on the basis of the trial results and the scope of the trial (including the number of patients)	

^a^Including the MEB’s advice on N-of-1 methodology in general, given during the design stage of the ephedrine trial before the data were available

^b^For rare diseases an applicant may propose and motivate (and discuss with the MEB) which level of evidence they would consider sufficient, e.g., increase type I error to 10% instead of 5% and/or register with a small sample (because the disease is very rare, mechanism of action well-understood and generalizable)

^c^Applicants should present a discussion of why the treatment effect could be generalized to the population intended. This should address whether the included patients are sufficiently diverse (patient characteristics, context of care surrounding the patients)

^d^Dealing with several N-of-1 trials is like dealing with a meta-analysis with patients instead of trials. Therefore, as an analogue of a Forest plot, a box-and-whisker plot should be provided. Per patient, this will provide information on the median (and mean) effect, the quartile range of effects, and full range of effects seen for that patient. This will provide information on the generalizability (boxes closer together: more uniform effect over patients) as well as of the repeatability of the effect within a patient (smaller boxes, better repeatability)

^e^If heterogeneity of effects is substantial: a discussion of sources and explanation for this heterogeneity should be attempted. This should include differences in patients’ characteristics, their context (e.g., the surrounding care in the hospital) and treatment (is the treatment differentially implemented per patient). If given, the interpretability of an overall mean should be discussed. E.g. if treatment effects vary much, the value of an overall mean has no interpretation. However, a large variation of effects but all “positive” supported by a significant overall test could support the statement that the treatment works (with differential effect)

#### Suitability of the N-of-1 design and presentation of data

NHCI stated that the Feasible Information Trajectory [24] indicates that for the indication ephedrine as add-on therapy for myasthenia gravis patients responding insufficiently to usual treatment, the following study characteristics are required for demonstration of effectiveness: randomisation, inclusion of a control group, and blinding of patients and physicians. These characteristics are all feasible in the indication at hand, but a limiting factor is the small size of the patient group. The NHCI concluded that N-of-1 trials are eligible for the consideration of the reimbursement of ephedrine for the indication at hand. Furthermore, the usual methodological provisos for N-of-1 trials apply (chronic disease, symptomatic treatment, etc.).

A further condition for the NHCI to consider N-of-1 trials as evidence for reimbursement is that the results of individual N-of-1 trials from a series must be aggregated, to enable a pronouncement on effectiveness at the population level. Trial design requires consideration of the number of measurements per patient and the total number of patients to be included. Finally, the NHCI only considers evidence after peer-reviewed publication. Taken together, published evidence from aggregated N-of-1 trials could be considered for reimbursement decisions on methodologically suitable, rare indications.

The MEB thought the N-of-1 design could be acceptable as proof of efficacy, under similar methodological provisos as mentioned by the NHCI. The MEB saw the N-of-1 design as a last resort option for rare conditions and limited to a particular group of products. This proviso was worded more strongly than in the NHCI’s advice. In contrast to the NHCI, the MEB actually judged whether the trial *results* showed that the methodological provisos for N-of-1 trials had been met (see section Variability of observed effects). While the MEB did not explicitly say that trial aggregation was required, this is implicit in the remark about numbers of patients crucial for generalization of the effect. Yet the MEB’s advice during the design stage of the trial suggests that statistical aggregation might not necessarily be required for assessing series of N-of-1 trials (“*If* given, the overall interpretability of an overall mean should be discussed”).

The MEB did not mention peer-reviewed publication as a requirement for evidence, not surprisingly as licensing authorities are used to considering unpublished data provided by drug manufacturers (sometimes supported with peer-reviewed publications). Importantly, the MEB noted that the N-of-1 design principle does not allow substantiation of the risk within the benefit/risk evaluation in a marketing authorization (i.e., licensing). Safety needs to be substantiated otherwise.

#### (Strength of) the evidence from the trials

##### Patient characteristics

The NHCI felt that the usual care patients were receiving alongside trial medication was suitable, as it was in accordance with an international guideline. The fact that one of the four patients was taking low-dose prednisone and an immunosuppressant during the trial led the NHCI to wonder how this might have influenced the results. The NHCI also wondered whether the results, all derived from females, are generalizable to males. A placebo comparator was considered suitable to the study question.

The MEB did not express an opinion on the clinical acceptability of the usual care received. It did note heterogeneity of the patients, not only in usual care but also in baseline scores and disease duration. It suggested that applicants should justify to what extent results based on four subjects can be extrapolated to the general MG population.

##### Comparator and primary outcome measure

The NHCI found the placebo comparator suitable for the question at hand. It also noted that the primary outcome measure was internationally endorsed. It advised providing further details on the measure’s validation history and its relationship to the EQ-5D instrument for quality of life, should the EQ-5D be unsuitable for this indication.

NHCI wondered whether a 5-day treatment period is sufficient to show a clinically relevant effect on the chosen outcome measures, considering that previous studies in myasthenia gravis used 14-day treatment periods. The MEB did not comment on the comparator, outcome measures or timing of measurements, but it should be noted that it provided helpful comments on these aspects when the trial protocol was under development.

##### Variability of observed effects within trial patients

The MEB, but not the NHCI, included in its advice judgement on whether the trial data supported the use of the N-of-1 design. It characterized the scores of individual patients’ placebo periods and ephedrine periods as “highly variable” and noted that “a clear, consistent treatment effect within a patient was not observed”. The MEB further commented that “within each patient, responses for the different scales [the primary and various secondary outcome measures] were not consistent, adding to the variability of the data, hampering the interpretation of the results”. The MEB also noted that individual patients could not be classified as responders. Note that the trial protocol had not defined criteria for “responders”.

##### Clinical relevance of the effect in the primary outcome measure

Both the NHCI and MEB questioned the clinical relevance of the found effect of ephedrine, 1 point reduction in QMG. The trial protocol had postulated a reduction of 3.5 points for the sample size calculation, based on previous trials which assessed immunosuppressive drugs. The NHCI would have found it helpful if the effect had also been expressed as a standardized mean difference. The MEB noted that clinical relevance should be discussed in terms of the benefit/risk balance. Both the NHCI and MEB pointed out that the trial performed was not suitable for testing the hypothesis that ephedrine postpones the use of corticosteroids. This would require a different outcome measure.

##### Sufficiency of the evidence for a decision

The NCHI stated that at the present stage, on the basis of the trial results and the scope of the trial (including the number of patients), it could not make a decision under the framework of “Established medical science and medical practice” on whether ephedrine is reimbursable for the indication under consideration. NHCI considered it the applicants’ responsibility to demonstrate that the statistical methodology as applied to this aggregated N-of-1 trial design is the correct methodology to enable a reliable statement about an effect at the population level. Interestingly, this result means that the research question on the desired level of precision for the trial turned out not to be within the remit of the NHCI’s framework.

The MEB stated that a decision on licensing could not be made on the basis of the trial data. It considered the current study design as failed, for reasons which it could not disentangle: heterogeneity of the study population, limited efficacy or unsuitability of the N-of-1 design for the indication in question. It suggested the following options: (1) inclusion of a “positive control” medication with a clear symptomatic effect in combination with selection of a more responsive patient population, (2) revision of the inclusion criteria of the study population to assure a constant disease activity and (3) a different study design e.g., a parallel group trial with a longer treatment duration where the day to day variability in scores can be averaged. The MEB, like the NHCI, did not advise how many more patients should be included in an aggregated N-of-1 trial to enable a decision.

#### Recommended regulatory route for getting ephedrine-on label for myasthenia gravis

The applicants asked the MEB what the best route would be for getting ephedrine on-label for myasthenia gravis, on the basis of ephedrine tablets imported from Spain or a product to be compounded in the future by a Dutch GMP-certified pharmacy. The MEB advised that three main routes exist: national authorization (with two variants), the mutual recognition procedure and the centralized procedure. Also, a post-approval registry would probably be needed due to the rarity of the disease and the uncertainties expressed in the MEB’s advice. Any route to marketing authorization would lead to additional costs and would demand specific knowledge on regulatory guidelines. Hence the MEB advised the applicant to look into options with a consultancy or a sponsor to facilitate the marketing authorization of ephedrine tablets for the indication MG. The next three subsections show MEB’s advice on possible regulatory routes to market authorization.

##### National authorization

It is possible to apply for a marketing authorization in the Netherlands either with a product developed oneself or with a product developed by a third party. Requirements for data substantiating a proposed product and relevant assessment depend on the potential legal basis. In general, marketing authorizations for medicinal products can be granted under different legal bases.

#### Application according to article 10(1) or 10(3) of Directive 2001/83/EC (generic/hybrid)

In case the company considers a generic legal basis as most appropriate, the proposed indication should be in line with that of the reference product. As ephedrine tablets are not authorized in the EU for the indication add-on treatment for myasthenia gravis to the knowledge of the MEB, the legal basis would be a hybrid application.

It is noted there are legal requirements on the suitability of a possible reference product. It is required that the marketing authorization of the reference product is granted in accordance with the Acquis Communautaire. Moreover, the legal basis, data exclusivity and market protection of the reference product should be taken into account.

#### Application according to article 8(3) of Directive 2001/83/EC (full dossier)

In case the company (applicant) chooses a 8(3) legal basis, a full dossier should be submitted for the proposed product. Guidance is available for a so-called “full-mixed” application which means the submitted dossier contains a combination of non-clinical and/or clinical studies and bibliographical references.

##### Mutual recognition procedure (MRP)

It is possible to perform a MRP with an existing marketing authorization (MA) for ephedrine tablets in other member states. A MRP is initiated by the marketing authorization holder of the existing MA in the relevant member state. This member state will act as a reference member state which will provide their assessment of the existing MA to the new member states at the start of the MRP.

In such applications, the legal basis and the indication should be in line with the existing MA. It is possible to add an indication via type II variation, either before or after the MRP. In this scenario, the MEB will be participating as a concerned member state and should also consider the indication of the existing MA as no ephedrine tablets are authorized in the Netherlands. In that case, the MEB will not have the lead in assessment of the future indication which is not their preference.

##### Centralised procedure (CP)

For this indication, a CP may be considered under the optional scope based on the interest of patients. Whether or not the proposed indication is considered to be an unmet medical need eligible for the CP will however need to be assessed by the EMA/CHMP. In case a CP is followed, a Paediatric Investigation Plan should be submitted. Of note: when applying for a CP, there is no difference in requirements for the content of the file and an application in accordance with article 10(3) is possible.

## Discussion

### Main findings

This study showed that implementing an N-of-1 trial protocol for an unlicensed drug for a rare indication was feasible for the trialists. The participating patients all finished the trial and considered it useful for themselves and others. The NHCI could potentially make reimbursement decisions using evidence from published, aggregated N-of-1 trials, in cases where the Fitting Evidence framework indicates that this design is indicated (e.g., add-on ephedrine for MG). For a licensing decision by the MEB, the N-of-1 design is a last-resort option for demonstrating treatment benefit in a rare disease. N-of-1 trials would likely have to be supplemented with other evidence on potential risk, in the context of benefit/risk assessment for drug licensing. If a drug is licensed in an EU country for one indication, several regulatory routes exist for getting it on-label for another indication in another country. Pursuing any of these routes requires regulatory expertise and entails costs.

Interestingly, various parties interpreted the results from the same series of N-of-one trials differently. Ephedrine was shown to be effective on the prespecified, main outcome measure inferred to the population level and expressed with a 95% confidence interval and *p* value. The trialists chose this traditional approach to facilitate interpretation at the regulatory level. A feature of the aggregated N-of-1 design is that it allows estimation of treatment effect both across individuals (i.e., at group level) and within individuals. This is usually considered a strength. For example, a paper by Senn, geared to non-statisticians, shows graphically and textually how the multiple cross-over design (equivalent to an aggregated N-of-1 design) identifies more sources of variation than the parallel group or single-crossover design [34]. Yet the inter- and intraindividual variability explicated in the ephedrine trial data seems to have undermined the MEB’s confidence in the effect estimate of the primary outcome measure. This is reflected in the MEB’s statements “On a group level, ephedrine tended to be effective but responses within patients were highly variable” and “a clear, consistent treatment effect within a patient was not observed”. It is also reflected in the MEB’s suggestion to consider instead doing “a parallel group trial with a longer treatment duration, where the day-to-day variability in scores can be averaged”. Surprisingly, a strength of the N-of-1 design (extra explicitation of variation) acted as a weakness for the MEB.

The effect of ephedrine was furthermore modest (1 point on the QMG scale). The trial protocol did not mention that for an add-on therapy, a lower threshold for clinical relevance could be considered than the 3.5 points (QMG) which had been used in earlier studies on (long-term) immunosuppressive treatment. A different hypothesis for benefit (postponing treatment with [higher doses of] corticosteroids) would require a new study with a new outcome measure, such as time to additional treatment.

### Strengths and weaknesses of the study

While many authors mention the suitability of the N-of-1 design for small patient groups, to our knowledge this is the first study which tested in practice whether evidence from N-of-1 trials is suitable for use in regulatory decisions. Another strength of our study was that the feasibility of a particular trial protocol and the utility of the evidence was examined from the perspective of multiple stakeholders. The study also provided an opportunity for regulatory agencies to test the consequences of policies which they had recently developed (for the NHCI, the Fitting Evidence framework [24] and for the MEB, plans to foster drug rediscovery [7]).

The study has limitations regarding generalizability. Results in the Netherlands, especially concerning reimbursement, are not directly applicable in other jurisdictions. Moreover, even within the Netherlands, the feasibility and utility of an N-of-1 trial protocol for a single condition and a single treatment may not be generalizable to N-of-1 trial protocols for other conditions and treatments. Fortunately, another research group in the Netherlands is also undertaking an aggregated N-of-1 trial for an unlicensed treatments for a rare disease [35].

The study also has limitations in the extent to which stakeholders were attuned. The briefing document submitted to the regulatory agencies was modelled on reimbursement dossiers, not licensing dossiers, yet the MEB was asked about the sufficiency of the evidence for licensing. It was therefore somewhat premature to ask this question, and the MEB’s advice was limited to general points on dossier requirements, e.g., that N-of-1 trials are not sufficient to substantiate risk. In fact safety might be substantiated with safety data from the literature, experience of other patient groups who use the drug for other indications, and open label extension studies for the indication in question.

### Strengths and weaknesses in relation to other studies

The utility of N-of-1 trials has been addressed in several other studies. In a narrative review, Lillie et al. note that N-of-1 trials are suitable for rare diseases and propose that they be used for treatment repositioning [10]. Their paper also provides arguments why both aggregated N-of-1 trials and traditional RCTs can provide evidence at the population level, albeit via different routes. More recently, Shamseer et al. noted that if evidence from N-of-1 trials is reported according to CENT standards (an N-of-1 specific extension of the CONSORT standards), it may “be of use to regulators when making decisions about additional conditions of use for particular treatments” [17]. While these studies respectively provide theoretical insights and new reporting standards (which were considered in the preparation of our Background Document), our research adds empirical evidence about the challenges of using evidence from N-of-1 trials at the regulatory level.

Nikles et al. studied the feasibility and usefulness of N-of-1 trials from multiple perspectives, by interviewing representatives of various Australian stakeholder groups including health care, clinician and patient organisations [21]. The interviewees had varying degrees of familiarity with N-of-1 trials, in contrast to our study which studied responses to a concrete trial. Although in the Nikles study stakeholders mentioned utility of N-of-1 trials in conjunction with reimbursement, this always seemed to be in the context of expensive drugs or drugs with contested cost-effectiveness. Possibly, N-of-1 trials are not needed in Australia for inexpensive unlicensed treatments for rare diseases, if treatments of that type are more accessible than in the Netherlands [21].

Larson et al. described the feasibility of N-of-1 trials in relation to a service which facilitated N-of-1 trials at the University of Washington over a 2-year period [36]. An important difference with our study is that the service aimed at supporting therapeutic decisions for many referring physicians and for a broad range of conditions and treatments, rather than aggregating evidence at the population level for a single condition and treatment. Forty trials were commenced and 34 were completed (85%), indicating a rather high completion rate, similar to our study (100%). In the Larson study, each trial had a unique protocol, but almost half of the completed trials (14/34) had six treatment periods of 1 to 2 weeks, suggesting comparability to the ephedrine trial. For the trial service, each trial cost roughly $400 to $500 1990 U.S. dollars for clinical, administrative and research activities, though the authors state that that is probably an underestimate. The ephedrine trial was much more expensive when calculated per patient. The trial service in Washington may have been cheaper through economy of scale (for example the trial service had implemented an expedited route for IRB evaluation of each trial protocol), as well as the fact that the ephedrine trial included implementation activities and preparation of a dossier for regulatory agencies. The study by Larson et al. presented quantitative outcomes of patients’ views on feasibility (e.g., 32% of patients “felt it was fun to try and guess which medication they were taking”). Items mentioned in the Larson study provided input for the interview guide in our study, which through its qualitative design explored how and why patients attached importance to various aspects of feasibility and utility (e.g., how and why guessing the trial medication was perceived in a variety of ways).

### How trialists can improve feasibility of N-of-1 trials from patients’ perspective

This study suggests that for trialists, performing aggregated N-of-1 trials is certainly feasible in a framework of clinical research. Patients found the trial useful and generally feasible, and made suggestions to improve feasibility. As soon as one patient expressed concern about the time it took to speak to the trial physician, measures were taken to facilitate contact for the patients still in the trial. The rest of this paragraph discusses how other suggestions by patients (shown in italics) could be implemented in any future N-of-1 trials. *Estimate beforehand whether patients can potentially handle disappointment, if they happen to compare experiences with fellow trial patients.* This would be hard to implement directly, but patients could be informed beforehand that their trial might be scheduled in parallel with trials of other patients, with adjacent appointments. *Perform weekly measurements in each patient’s home town.* This would create extra heterogeneity, which is undesirable in an aggregated trial with a small group. Training professionals in various towns is not feasible for a small trial. If a validated outcome measure existed which patients could report themselves, less travel would be necessary, but this is currently lacking for ephedrine in myasthenia gravis and may also be an issue for other rare indications. Lack of a validated patient reported outcome measure about energy was in fact the obstacle for implementing another patient’s suggestion: *include an item about experienced energy in the questionnaire. Avoid breaks in the trial to make the outcome clearer.* Concerns about the validity of prolonged N-of-1 trials is to some extent justified. Ideally, at the start of an N-of-1 trial, the disease should be in a stable phase. This is not always the case with myasthenia gravis over longer periods of time. *Make capsules with trial medication smaller, for easier swallowing.* The size of the capsule was determined by the size of the ephedrine tablets. A smaller sized capsule might be possible if a different form of ephedrine had been available or capsules would have to be made out of raw material. The advantage of using existing tablets in cases like this small trial with limited funding is that no manufacturing or extensive analysis has to be performed.

### Implications of the study for reimbursement

This study suggests that no fundamental obstacles exist for the Dutch reimbursement authority to make a decision on the basis of evidence gleaned from a small series of N-of-1 trials, supported by relevant literature. The evidence must be published and, in the case of ephedrine, further support is needed for the clinical relevance of the treatment effect. On the one hand this could be addressed by expressing the effect size in a standardized way (e.g., standardized mean difference) and by clarifying the effect’s relationship to a suitable measure of quality of life, preferably EQ-5D. It should be noted that NHCI does not have a threshold for effect size nor does it have an absolute requirement for EQ-5D data, except that for cost-effectiveness evaluation applicants must provide justification if they do not report EQ-5D [37]. Evaluation of cost-effectiveness is unlikely to be an issue for inexpensive treatments for small patient groups, as the Netherlands has an exemption from pharmacoeconomic evaluation for outpatient medicines with annual budget impact less than 2.5 million Euros. Still, if only a modest effect of ephedrine can be shown in short term trial trials, a decision to reimburse may require evidence gathered over a longer period of time and with a different design than N-of-1, to test whether ephedrine reduces the use of drugs with a riskier profile such as prednisone. Thus, proving effectiveness of a moderately priced drug may require more expensive studies than the current trial.

A potentially resolvable issue is the NHCI’s advice that previous studies in MG used 14 day treatment periods, as compared to the 5-day treatment periods in our trial. This would however lengthen the trial for individual patients and therefore lower the chance of a stable disease course during the trial.

### Implications of the study for licensing

In contrast to the results on reimbursement, this study suggests that getting a drug like ephedrine “on license” affordably is a greater challenge. To prepare a dossier for market authorisation, academic researchers would need to involve experts in regulatory affairs, for example from a pharmaceutical company or consultancy firm. This investment might have to be recouped through raising the price of the drug. In the process, evidence from aggregated N-of-1 trials would have to be supplemented with data from other sources to demonstrate safety. For rare diseases and moderately priced drugs, a role for public institutions (e.g., universities) or other non-commercial parties is conceivable. This might contribute to accessibility and sustainability of the drug market in the age of personalized medicine.

Interpreting aggregated N-of-1 data on treatment effect may also be a barrier for market authorization. The explicitation of several levels of variation, a positive feature of the aggregated N-of-1 design, may distract from a main outcome measure even when it is expressed in traditional terms (population level effects, 95% confidence interval, *p* value). In the era of personalized medicine, agreement is urgently needed on the interpretation of data from study designs geared to small groups. Furthermore, the MEB’s advice on consistency of the evidence, heterogeneity of the trial patients and the stability of the disease could be relevant for any small, aggregated N-of-1 trial designed for regulatory purposes, hence these issues are detailed below.

#### Consistency of the evidence

The MEB advised that “within each patient, responses for the different scales were not consistent, adding to the variability of the data, hampering the interpretation of the results”. It is unclear by what standards the MEB judged the consistency of these scales. The secondary outcome measures all showed a statistically significant effect of ephedrine at the prespecified, aggregated level of trial patients (Briefing document, Table two [Additional file 1]). During the discussion meeting the MEB had remarked that graphing of individual patient data did not resemble a saw-tooth pattern in individual patients (e.g., consistently up during placebo and consistently down during ephedrine periods). However, Huber et al. [38] have summarized disadvantages of visual analysis of N-of-1 trial data, including lack of standardization and poor inter- and intrarater reliability [38]. Another issue, probably related to the MEB’s view on the inconsistency of the evidence, is its observation that “individual patients could not be classified as responders”. It is not clear why this should be an issue, since the trial was not powered at the individual level but rather at an aggregated level. A recent, didactic paper by Araujo et al. [39] addresses variation in sets of N-of-1 trials; our analysis method was in line with the recommendations in that paper.

#### Heterogeneity of patients

It seems surprising that the MEB advised that heterogeneity in baseline scores and disease duration might limit the extrapolation of trial findings to the general MG population. It is not clear how this concern might be addressed, for three reasons: 1) the proposed indication for ephedrine was not defined by disease duration, 2) heterogeneity of usual care in the trial patients was within the predefined inclusion criteria and 3) baseline score in the primary outcome measure was not an inclusion or exclusion criterion. Thus it is unclear how heterogeneity within the trial patients might bias the external validity of the results. This discussion illustrates a different starting point for discussions between physicians treating rare disease patients and regulatory authorities. The first look for a treatment for their patients, the second start from the treatment. For a proof-of-principle study homogeneous patients would be preferable.

#### Disease stability

One of MEB’s suggestions was to “revise the inclusion criteria of the study population to assure a constant disease activity [which might] increase the probability to show a symptomatic treatment effect”. A criterion for applying the N-of-1 design is disease stability. It is true that the severity of motor symptoms in MG may vary in the course of the day, for example in the case of exercise. The severity of symptoms can also change over longer stretches of time as part of the natural history of the disease. However, the design of the study enabled estimation of the variability of disease severity (i.e., severity under placebo) using multiple measurements, during a 6-week period, under the assumption of equal variance among patients (as prespecified in the protocol). Thus the authors’ view is that it still might be reasonable to consider myasthenia gravis stable enough for studying short-term effects with an N-of-1 design.

The MEB also suggested how to show that ephedrine for myasthenia gravis meets the conditions for an N-of-1 trial: “inclusion of a positive control with a clear symptomatic effect (e.g., N-of-1 one trial with placebo, ephedrine and acetylcholinesterase inhibitor treatment periods) in combination with selection of a more responsive patient population. The first suggestion makes sense for N-of-1 trials where patients are not dependent on the positive control as a concomitant medication, i.e., as a proof of concept in drug naïve patients. However, the authors who are experienced in treating MG believe that it would be unethical to withhold acetylcholinesterase inhibitors from patients who are experiencing mild to moderate symptoms. Regarding the second suggestion, to include a more responsive population, it is currently not possible to implement for MG because it is unclear which patient characteristics are associated with responsiveness to ephedrine.

## Conclusions

Ephedrine is an inexpensive drug (less than €1000 per patient per year) which is potentially useful for a very small patient population (about 40 in the Netherlands, personal communication (JJGMV)) under treatment in tertiary centers. An aggregated N-of-1 trial with four patients showed a statistically significant treatment effect of the main outcome measure at the population level. However, the effect was smaller than the minimal clinically important difference based on the literature. Further research is needed to justify the clinical relevance of the treatment effect of ephedrine, for example an RCT comparing ephedrine and placebo with the primary outcome time to (high dose) corticosteroid use or cumulative corticosteroid use over time. Notwithstanding, this study suggests that there are no fundamental barriers for the NHCI to make reimbursement decisions using evidence from aggregated N-of-1 trials with small numbers of patients. Formal applications to the NHCI are needed to test whether the NHCI actually will make positive reimbursement decisions for unlicensed or off-label drugs for small patient groups, using aggregated data from N-of-1 trials, for ephedrine for MG as well as other drugs for other diseases. At present the reimbursement status of ephedrine for MG is still unresolved. Indeed, for the two patients continuing after the open-label extension, one health insurance company agreed to reimburse ephedrine while another denied reimbursement.

This study suggests that current drug licensing frameworks are not conducive to getting treatments for small groups “on label” affordably. More research is needed on better business models. The study also suggests that within current licensing frameworks, the benefit of drugs for small populations could be assessed using N-of-1 trials, provided that the condition and treatment meet the methodological criteria for N-of-1 trials. In the regulatory exploration of whether the N-of-1 design is suitable for ephedrine for myasthenia gravis, it emerged that individual variability can cause tension in the assessment of population effects. Further research is needed to explore whether the explicitation of variability, as in aggregated N-of-1 trials, is perceived as undermining the strength of evidence for effectiveness at the population level. Several urgent questions remain for the “rediscovery” of affordably priced drugs.

## Additional file

Additional file 1: Briefing document. Pharmacotherapeutic value and benefit/risk aspects of ephedrine, for the indication: add-on therapy for myasthenia gravis. Briefing document as submitted to the National Health Care Institute and the Medicines Evaluation Board for scientific advice. Parts of the document were subsequently published as [29]. (PDF 1102 kb)

## Abbreviations

CENT: CONSORT extension for reporting N-of-1 trials
CHMP: Committee for Medicinal Products for Human Use
CONSORT: Consolidated Standards of Reporting Trials
COREQ: Consolidated criteria for reporting qualitative research
CP: Centralised procedure
EMA: European Medicines Agency
EQ5-D: European Quality of Life 5-Dimensions questionnaire
GMP: Good manufacturing practice
IRB: Institutional review board
LUMC: Leiden University Medical Center
MA: Marketing authorisation
MCID: Minimal clinically important difference
MEB: Dutch Medicines Evaluation Board (College ter beoordeling van geneesmiddelen)
MG: Myasthenia gravis
MGFA: Myasthenia Gravis Foundation of America
MRP: Mutual recognition procedure
NHCI: Dutch National Health Care Institute (Zorginstituut Nederland)
QMG: Quantitative Myasthenia Gravis test
RCT: Randomised controlled trial

## Footnotes

1 Rational pharmacotherapy is defined as the treatment, prevention or diagnosis of a disease using a medicine which is in a form suitable for the patient, whose effectivity and effectiveness have been shown in the scientific literature and which is the most economical for the health care insurer and the patient [40].
