# Supplementary material for: Aggregated N-of-1 trials for unlicensed medicines for small populations: an assessment of a trial with ephedrine for myasthenia gravis
Source: Orphanet J Rare Dis. 2017 May 12;12:88. doi: 10.1186/s13023-017-0636-y (PMC5427624; doi:10.1186/s13023-017-0636-y)
Supplement: Additional file 1: — Briefing document. Pharmacotherapeutic value and benefit/risk aspects of ephedrine, for the indication: add-on therapy for myasthenia gravis. Briefing document as submitted to the National Health Care Institute and the Medicines Evaluation Board for scientific advice. Parts of the document were subsequently published as [29]. (PDF 1102 kb) [file 13023_2017_636_MOESM1_ESM.pdf]

BRIEFING DOCUMENT
Pharmacotherapeutic value and benefit/risk aspects of ephedrine

For the indication:
Add-on therapy for Myasthenia gravis

Dossier status: **To be considered by Zorginstituut Nederland (for an internal report) and by College ter Beoordeling van Geneesmiddelen (for customised scientific advice)**

Date: 27 July 2015

Submitted by: **Prof. J.J.G.M. Verschuuren, Leids Universitair Medisch Centrum, Department of Neurology**

Contact person: Alexander F. Lipka
Leids Universitair Medisch Centrum
Dept of Neurology, J3-R-166
PO box 9600
2300 RC Leiden
T 071-5262118 / 5262197
A.F.Lipka@lumc.nl

Table of contents

ABBREVIATIONS **3**

SUMMARY **4**

1. INTRODUCTION **5**

1.1 MEDICATION 5
1.2.A REGISTERED INDICATION(S) OF THE MEDICINE 6
1.2.B INDICATION TO BE ASSESSED 7
1.3 CURRENT REIMBURSEMENT STATUS 7
1.4 ETIOLOGY OF THE DISEASE 7
1.5 SYMPTOMS OF THE CONDITION 7
1.6 PREVALENCE/INCIDENCE 8
1.7 SEVERITY OF THE DISEASE 8
1.8 TREATMENT OF THE DISEASE 8
1.9 PROPOSED INDICATION AND TREATMENT SCHEDULE 9

2. METHODS **11**

2.1 COMPARATOR 11
2.2 LITERATURE RESEARCH 11
2.3 RELEVANT CLINICAL TRIALS 11

3. THERAPEUTIC VALUE **15**

3.1 BENEFICIAL EFFECTS 15
3.1.1 OUTCOME MEASURES 15
3.1.2 MOTIVATION FOR EXCLUDING OUTCOME MEASURES 15
3.1.3 RESULTS OF THE PRIMARY AND SECONDARY OUTCOMES OF THE STUDY 15
3.2 ADVERSE EFFECTS 17
3.3 EXPERIENCE 19
3.4 APPLICABILITY 19
3.5 EASE OF USE 20

4. CONCLUSION AND CLAIM **20**

APPENDIX

Information on marketing authorization and registration of ephedrine tablets imported from Spain:

A1 Summary of product characteristics
A2 GMP certificate of manufacturer
A3 documentation from AEMPS/CIMA

SUPPLEMENTAL FIGURES

S1 Flow Diagram of recruitment, follow-up, and analysis
S2 Mean treatment effect per cycle for each patient
S3 Individual mean scores during ephedrine and placebo treatment
S4 Cardiovascular effects of ephedrine compared to placebo.

Abbreviations

AChI	Acetylcholinesterase inhibitor
AChR	Acetylcholine receptor
CBG	College ter Beoordeling van Geneesmiddelen
CI	Confidence interval
ECG	Electrocardiography
EudraCT	European drug regulatory affairs Clinical Trials
ICU	Intensive care unit
IVIG	Intravenous immunoglobulin
LUMC	Leiden University Medical Center
METC	Medisch Ethische Toetsingscommissie; institutional ethical review board
MG	Myasthenia gravis
MG-ADL	Myasthenia Gravis-Activities of Daily Living profile
MGFA	Myasthenia Gravis Foundation of America
MuSK	Muscle-specific kinase
NMJ	Neuromuscular junction
Ph.Eur.	European Pharmacopoeia
QMG	Quantitative Myasthenia Gravis score
QTc	Corrected QT-interval
SmPC	Summary of Product Characteristics<br>(in Dutch: officiële productinfomatie IB1-tekst)
Tmax	Time of estimated maximum serum concentration
VAS	Visual analogue scale
ZIN	Zorginstituut Nederland

Summary

Claim of the applicant

Aggregated data from a series of N-of-1 trials showed that ephedrine is an effective treatment for patients with myasthenia gravis who do not respond sufficiently to first line treatment but for whom high dose immunomodulatory or immunosuppressive treatment might be too early or lead to severe side effects. The effect of ephedrine is modest but consistent, and clinically relevant because it may postpone or prevent use of treatments with a higher risk profile. For patients without a contraindication for ephedrine, the risks of ephedrine treatment are acceptable as shown in the series of N-of-1 trials, and a systematic literature review. Ephedrine is already licensed in Spain, for a different indication.

Introduction

Myasthenia gravis is a rare autoimmune neuromuscular condition which initially responds favourably to symptomatic treatment with acetylcholinesterase inhibitors (AChIs) that act on the neuromuscular junction (NMJ). Second line treatment usually consists of high doses of prednisone, either alone or in combination with immunomodulating or immunosuppressive drugs, which may have serious side effects. A potential alternative is the short-acting drug ephedrine which – together with AChIs or low-dose prednisone - may abolish or postpone the need for high-dose immunomodulating or immunosuppressive therapies.

Ephedrine is a sympathomimetic agent which mainly acts on the adrenergic receptors. In this briefing document, we will summarize current evidence for risks and benefits of ephedrine as add-on treatment for patients with myasthenia gravis.

Methods

Our research group has performed a series of randomized, double blind, multiple crossover N-of-1 trials in four patients. Ephedrine added onto usual care, i.e. treatment with pyridostigmine and/or low dose immunosuppressants, was compared with add-on placebo treatment in three crossover cycles consisting of both treatment arms. The primary outcome was the Quantitative Myasthenia Gravis (QMG) score, which was designed specifically for myasthenia gravis and quantifies muscle strength and fatigability. No other RCTs to determine the effect of ephedrine for myasthenia gravis have been performed.

Therapeutic value

Beneficial effects: Add-on treatment with ephedrine compared to placebo improved QMG score by 1.0 point (95% confidence interval 0.21-1.79) in the trial patients. The population treatment effect was also statistically significant. Ephedrine also showed a significant trial average treatment effect for all secondary outcomes, improving MG-Composite by 2.7, MG-ADL by 1.0 and VAS score for muscle strength by 1.1.

Adverse effects: Adverse events were all mild and included palpitations, tremor and

restlessness. Although all ECGs were normal, ephedrine prolonged the corrected QT interval, within the normal physiological limits.

Experience: limited case descriptions are available in the literature.

Applicability: A small subset of myasthenia gravis patients with moderate disease severity are expected to benefit from add-on treatment.

Ease of use: a standard dose of ephedrine 2dd 25mg in small tablets is recommended.

Conclusion

Ephedrine as add-on treatment for myasthenia gravis resulted in a modest but consistent improvement of symptoms and weakness. In MG patients with moderate severity, this treatment could prevent necessity of more aggressive treatment options.

1. Introduction

1.1 Medication

Pharmaceutical form: ephedrine tablets

Method of administration: oral

Dose: 25 mg 2 times daily

Composition: Ephedrine HCl (API), Excipients: microcrystalline cellulose, talc, magnesium stearate and carboxymethyl starch.

Manufacturerer: Laboratorios ERN S.A., Barcelona, Spain

Purity: not applicable. For the clinical trial, tablets with a marketing authorisation (see below) were used. The tablets were divided in half. The divided tablets complied with the European Pharmacopoeia (Ph.Eur.) test for uniformity of mass of subdivided parts.

Quality control: QP released by Laboratorios ERN. Laboratorios ERN has a marketing authorisation (Eudra GMP nr 0152), the ephedrine tablets are registered in the Spanish register (see section 1.2.a).

Mechanism of action

Ephedrine is a sympathomimetic agent which mainly affects the adrenergic receptors.[1] Its mechanism of action in myasthenia gravis (MG) has been investigated, but is not completely understood.[2-7] Ephedrine is a drug that is only used by a small number of patients with myasthenia. Clinically, these patients seem to benefit from this drug.[7;8]

Three possible modes of action are likely to play a role.
1. An acute effect on the neuromuscular junction. For example, Sieb and Engel measured the effects of different concentrations of ephedrine on the muscle endplate in

an in vitro canine model using microelectrode techniques. Ephedrine increased the quantal content of the endplate potential as well as the probability of quantal release, but at concentrations that would not be reached in patients.[5] Possibly, ephedrine improves muscular transmission by stimulating beta-2-adrenergic receptors, as well as by partially stabilising the structure of the neuromuscular junction.[7] There is no definitive evidence regarding the onset of the effect of treatment, but clinical experience (JJGMV) and isolated case reports suggest a noticeable effect within hours to days in autoimmune myasthenia gravis.[9]

2. A chronic beneficial effect on muscle. In some patients with a hereditary form of myasthenia (congenital myasthenic syndromes), it has been reported that the maximum effect of ephedrine was only reached after several weeks to months of treatment.[7;10]

3. A beneficial effect on the fatigability of muscles, which could in part be due to the stimulating effect of ephedrine on the central nervous system.[11;12]

<u>Pharmacokinetics, side effects and contraindications</u>

Ephedrine is rapidly absorbed. It is partially metabolised to norephedrine in the liver, but is mainly excreted in the urine unchanged.[13] The renal clearance is dependent on urine pH. Mean plasma half-life is approximately six hours and ranges from three to 11 hours.[1;13] The most common side effects are tachycardia (rapid heart rate), anxiety, nausea, restlessness and insomnia. In children, ephedrine may stimulate nocturnal enuresis, sleeplessness, or sometimes sedation.[1;7] Contraindications for ephedrine are renal insufficiency, hypertension and certain cardiac diseases, such as long QT syndrome and angina pectoris.[1;13]

1.2.a Registered indication(s) of the medicine

In Spain, ephedrine tablets are registered for treatment of asthma and hay fever. In the Netherlands, ephedrine is only available as a solution for injection, for bronchospasm or perioperative hypotension.

No oral ephedrine preparation is currently registered for marketing approval in humans in the EU (EMA 2011) for myasthenia gravis, and pharmacies must either import the tablets from Spain (as used in the N-of-1 trials) or compound the ephedrine tablets themselves.

In Appendix A2 and A3, information is provided concerning the marketing authorisation and registration of the Spanish tablets.

1.2.b Indication to be assessed

Ephedrine is proposed as an add-on therapy to pyridostigmine monotherapy or the combination of pyridostigmine and low-dose immunosuppressive or immunomodulatory drugs in adult patients with generalized autoimmune myasthenia gravis who do not respond sufficiently to standard treatment.

1.3 Current reimbursement status

When neurologists in a Dutch academic medical center prescribe ephedrine, it cannot be reimbursed as specialized medical care (medisch specialistische zorg) because the patients take the tablets at home. Reimbursement currently depends on case-by-case decisions of various health insurance companies. Zorginstituut Nederland has not (yet) been asked to advise whether ephedrine as add-on treatment for myasthenia gravis is 'rational pharmacotherapy' for a rare indication, within the reimbursement framework for extramural medicines.

1.4 Etiology of the disease

Myasthenia gravis (MG), ICD-10 code G70.0, is a rare disorder in which autoimmune antibodies are directed against components of the neuromuscular junction, which results in an impairment of neuromuscular transmission, and thus muscle weakness. In some patients, myasthenia gravis is associated with the presence of thymoma.

1.5 Symptoms of the condition

The presenting symptom is often fatigability and weakness of the ocular muscles, resulting in ptosis and diplopia. In 80% percent of cases, this weakness generalises to the rest of the body, with proximal muscles often more affected than distal ones.[14] Symptoms of generalised myasthenia gravis include fluctuating muscle weakness and muscle fatigability of the oropharyngeal muscles (resulting in dysarthria, dysphagia, and an expressionless face or "myasthenic sneer") and skeletal muscles, for example, the respiratory muscles (which may lead to a life-threatening myasthenic crisis) and neck muscles ("dropped head sign"). Muscle weakness and fatigability are often more pronounced at the end of the day, after exercise, during warm weather or during illness.[15;16]

Diagnosis of MG is based on:

- Typical pattern of fluctuating muscle weakness suggestive of myasthenia gravis

  and at least 1 abnormal additional investigation

- A positive serologic test for acetylcholine receptor (AChR) or muscle-specific kinase (MuSK) antibodies, or
- Electrophysiological evidence: presence of at least 10% decrement upon low rate repetitive nerve stimulation, or increased jitter and blocking upon single fibre electromyography
- Neostigmine testing: short-term improvement of muscle weakness after injection of short-acting acetylcholinesterase inhibitors

Serological evidence of the diagnosis (esp. AChR antibodies) can be found in > 85% of patients, facilitating confirmation of the diagnosis.

1.6 Prevalence/incidence

International literature suggests a prevalence of MG of approximately 78 per million, with a range of 15 to 200 per million.[17-19] A recent epidemiological study in the Netherlands shows a point prevalence of 167 per million.[20] The annual incidence reported in the international literature varies widely, between 1.7 and 10.4 per million, with an annual incidence of 6.48 per million in the Netherlands.[14;21]

During the recruitment phase of the trial, only 5-10% of patients in our tertiary, academic clinic were eligible for the clinical trial investigating the effect of ephedrine. Many patients had either too mild or too severe disease, or had contra-indications to ephedrine. This percentage is likely to be even lower in non-academic centres, where more relatively mild patients are under treatment. Therefore, it is estimated that 50-100 Dutch patients at any given time are eligible for add-on, second line treatment with ephedrine. The indication is therefore rarer than 1:150,000 a criterium for reimbursement of rational use of a non-registered medicine.[22]

1.7 Severity of the disease

Myasthenia gravis is a potentially life-threatening disease, and despite the availability of multiple treatment options an increased mortality compared to the general population has been reported.[23] A majority of patients develop generalized muscle weakness and about 5-10% of patients develop respiratory muscle weakness, possibly resulting in intubation and ventilation in the ICU.[14] Quality of life is reduced in patients with myasthenia gravis, especially in patients with more severe disease.[24;25] This reduction was reported for both physical and mental aspects of quality of life. The strongest predictor of quality of life was a generalized disease course, which defines the subgroup of myasthenia gravis likely to benefit most from additional treatment options including ephedrine. Patients with myasthenia gravis report many limitations in activity of daily life, including talking, several problems with food intake, as well as limitations in mobility.[26]

1.8 Treatment of the disease

First line treatment of myasthenia gravis consists of acetylcholinesterase inhibitors (AChIs) and thymomectomy, if thymoma is present. Acetylcholinesterase inhibiting medicines, such as pyridostigmine (Mestinon®), inhibit the enzyme acetylcholinesterase, which is responsible for hydrolysing the neurotransmitter acetylcholine and thus inhibits neuromuscular transmission. By inhibiting the activity of this enzyme, AChIs prolong the activity of the neurotransmitter acetylcholine and enhance neuromuscular transmission.[16;27] Most patients initially respond favourably to treatment with acetylcholinesterase inhibitors and it is well tolerated by most patients. There are several different types of acetylcholinesterase inhibitors, but in clinical practice only pyridostigmine is used for long-term treatment purposes (Mestinon®, or the slow release preparations Mestinon retard® or Timespan®).[16]

If patients no longer respond favourably to pyridostigmine, the second line of treatment consists of immunosuppressive treatment using high doses of corticosteroids (prednisone), most often in combination with azathioprine.[16;27] These therapies may lead to a complete remission of MG, but can also have serious side effects. For example, well known side effects of prednisone treatment include diabetes mellitus, osteoporosis, cataracts, Cushing syndrome and aseptic osteonecrosis (KNMP 2012). Rare side effects of azathioprine include bone marrow suppression, leukopenia and opportunistic infections (KNMP 2012).
In a series of 126 AChR MG patients from the LUMC 40% used only pyridostigmine, while 60% needed prednisone or prednisone in combination with another

immunosuppressive drug to control their disease

A guideline from the European Academy of Neurology (EAN, previously EFNS), among others in collaboration with our research group, is available for treatment of myasthenia gravis, in which the treatment options mentioned above are evaluated.[27] This guideline recommends symptomatic therapy with pyridostigmine for all MG patients, thymectomy for early-onset generalized patients and immunosuppressive medication for all patients with progressive MG symptoms. This is in line with the protocol of the Dutch Neuromuscular Research Centre (www.isno.nl) "Pharmacological treatment of myasthenic syndromes"

1.9 Proposed indication and treatment schedule

For patients who do not respond favourably to first line treatment with pyridostigmine. Current second line treatment usually consists of high doses of immunosuppressive or immunomodulatory drugs. Ephedrine is proposed as an add-on second line treatment to pyridostigmine and/or low dose immunosuppressive or immunomodulatory drugs. Below we detail the treatment schedule for MG, including the proposed subgroup of MG patients in whom add-on treatment with ephedrine is indicated.

Figure 1 Proposed treatment schedule

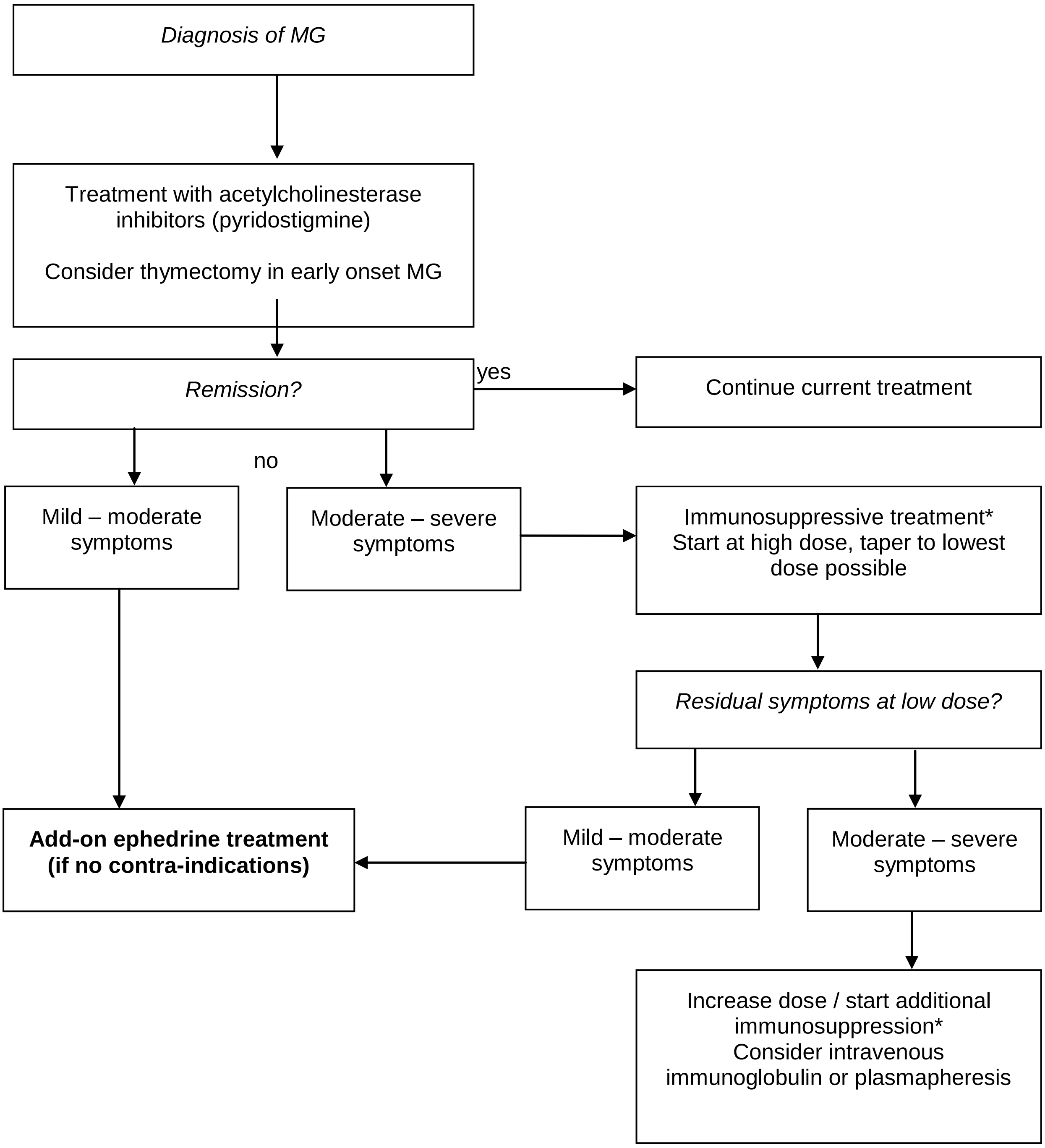

* Immunosuppressive treatment usually consists of prednisone starting at high-dose. Frequently azathioprine is started to reduce long-term dose of prednisone. Other immunosuppressants prescribed for myasthenia gravis include mycophenolate mofetil, cyclosporine, cyclofosfamide, rituximab, tacrolimus and methotrexate.

2. Methods

2.1 Comparator

Ephedrine added onto usual care, i.e. treatment with pyridostigmine and/or low dose immunosuppressants, is compared to placebo added on to usual care.

2.2 Literature research

We have conducted a Cochrane systematic review of the medical literature on the effects of ephedrine in myasthenia gravis.[8] Our review shows that no randomised controlled trials have been conducted to determine the effect of ephedrine in patients with AChR MG, but a number case reports suggest that it may be effective in some patients.[28-30]

2.3 Relevant clinical trials

Description of clinical trial

1st author and year of publication	*design [numbers ITT/PP]*	*patients*		*intervention and control*	*follow-up period*	*primary outcome*
		number	*characteristics*			
Lipka et al, in preparation.	Series of randomized, double blind, multiple crossover N-of-1trials (ITT 4, PP 4)	4	generalized MG, currently treated with pyridostigmine and/or low dose prednisone and/or other steroid-sparing immunosuppressive drugs	Add-on ephedrine vs add-on placebo	6 weeks (multiple crossover phase)	QMG score

2.3.a Lipka et al. (in preparation). Trial registered under EudraCT no. 2014-001355-23.

Methodology and study design: series of randomized, controlled, double blind, N-of-1 trials. Each patient was treated for three weeks with ephedrine and three weeks placebo add-on treatment in a randomized, double-blind N-of-1 trial. Treatment was administered in three treatment cycles, each consisting of 2 periods in which either ephedrine 50mg daily in 2 doses or placebo was administered for 5 days, followed by a 2-day washout period. This was followed by 5 days of the alternate treatment, again with a 2-day washout period. Treatment order was randomized for each patient individually, within each cycle (an example is shown in Figure 1). Randomization was performed for each individual N-of-1 trial by the hospital pharmacy. Patients and investigators were blinded to the treatment sequence until completion of an individual N-of-1 trial, after which the individual results were discussed and patients were asked to participate in an open label extension phase for 6 months. The trial was registered under EudraCT no. 2014-001355-23 and an extensive description of the protocol has been published.[31]

Outcome measures:
The primary endpoint was the effect of add-on therapy with ephedrine compared to placebo, based on the Quantitative Myasthenia Gravis (QMG) score.[32;33] The QMG score is a severity score for muscle strength and fatigability consisting of 13 items, each scored from 0 (normal) to 3 (severe weakness). This endpoint was assessed for all patients enrolled to determine the trial average treatment effect. Only in case of significant improvement, the population treatment effect would be assessed to determine generalizability to other MG patients. (See section Statistical analyses.)

Secondary outcome parameters were the MG-Composite, MG-ADL scores and a VAS score for subjective assessment of muscle strength, in a muscle group predefined by the patient.[26;34] Individual treatment effects were also assessed for all outcome measures. All tests were performed on day 5 of treatment periods, at a predefined time and interval after all medication.

Adverse events were monitored during each treatment arm using questionnaires, which included a list of known side effects of ephedrine, as well as screening blood tests and ECGs at the end of treatment periods. During the first treatment cycle, patients were admitted on the first day in both treatment arms, during which vital signs and adverse events were recorded, as well as ECGs at time of estimated maximum serum concentration (Tmax).

Treatment preference for either of the treatment arms was recorded for each treatment cycle. Blinding was assessed by recording presumed randomization sequence by patient and investigator after each treatment period.

Inclusion criteria:

- ❑ Adult patients with a diagnosis of generalised MG, based on clinical signs or symptoms suggestive of generalised MG and confirmed by a positive serologic test for acetylcholine receptor (AChR) antibodies
- ❑ No adequate improvement of myasthenic symptoms with pyridostigmine, either alone or in combination with low dose (max. 15 mg/day) prednisone or other immunosuppressive drugs
- ❑ Stable dosages of pyridostigmine, prednisone or other immunosuppressive drugs for at least 6 weeks prior to trial enrolment

Exclusion criteria:

- ❑ Purely ocular myasthenia
- ❑ Ephedrine was not tolerated in the past or is contra-indicated due to myocardial ischemia; cardiac arrhythmia; inherited prolonged QT syndrome or prolonged QT interval; angle-closure glaucoma; psychiatric treatment; hypertension; poorly regulated diabetes; prostatic hypertrophy; thyrotoxicosis; or relevant drug interactions (MAO inhibitors, alpha and beta blockers)
- ❑ Prednisone dose of >15mg/day, recent (< 3 months) or regular intravenous immunoglobulin (IVIg) or plasma exchange
- ❑ Myasthenic crisis in the past three months
- ❑ Thymectomy in the past six months, or scheduled to take place during the N-of-1 trial
- ❑ Inability to fill out the study questionnaires, be interviewed in Dutch, undergo the trial's test procedures, give informed consent, or any other reason which renders the patient unsuitable to participate

Statistical analyses

By repeatedly exposing the patients to active treatment and placebo, we will gain information about the treatment effect on the QMG within each patient in the study, and hence about the average effect as well. Based on previous observations in our clinic and an earlier clinical trial, we estimated that the standard deviation of repeated measurements of QMG within a single person is 2.95.[35] For our sample size calculation, we assumed a mean treatment effect of 3.5, as previous studies deemed such an improvement to be clinically relevant.[32;36] To allow for variation of the treatment effect between patients, we assumed the individual treatment effects to be random with mean 3.5 and standard deviation of 1. Under these assumptions, our sample size calculation (carried out by means of Monte Carlo simulation) showed a sample size of 4 patients (3 cycles per patient) would yield 77% power to detect a non-zero treatment effect on average among the patients enrolled in the trial at the two-sided significance level alpha=0.05.

To test for a non-zero average treatment effect on the primary and secondary outcome, we use a standard linear model. This model includes fixed factors for patient and treatment, and their interaction. Thus, the model allows for patient-specific intercepts and patient-specific treatment effects. Besides an estimate of the average treatment effect, this model also produces results for treatment effect in individual patients.

Although a significant treatment effect on average across the patients in the study is important, it cannot be generalized directly to the entire population of eligible patients. So, only if we find a significant result, do we proceed to test the more ambitious hypothesis that the treatment effect differs significantly from zero on average across the population of eligible patients. To this end, we will fit a linear mixed model for QMG score with fixed effects for treatment and patient, and also a random treatment effect. We will test the significance of the fixed treatment effect in this model at the 0.05 level. Since we test this population average treatment effect only if the trial average treatment effect is significant, the overall type I error probability does not exceed 0.05.[37]

All data analyses are performed using R Foundation for Statistical Computing (version 3.0.2 Vienna, Austria).

Patient inclusion, dates and baseline characteristics

We screened 14 patients with AChR MG for entry in the study, of which 5 did not meet the criteria for the trial and 4 declined to participate (Figure 1). We included 5 patients, one patient discontinued before the actual start of the N-of-1 trial and was replaced. All four remaining patients completed the n-of-1 trial (Table 1). One patient did not complete a treatment cycle, after which the cycle was excluded and replaced by an extra randomized cycle, as was defined before analysis took place and approve by the METC.

Baseline characteristics of the study patients are shown in Table 1.

Table 1. Baseline characteristics of study patients

	Patient 1	Patient 2	Patient 3	Patient 4
**Age**	33	46	53	35
**Gender**	F	F	F	F
**Disease duration**	6 yr	21 yr	7 yr	5 yr
**AChR titer (nmol/L)**	>5.0	>5.0	>5.0	>5.0
**QMG score**	7	8	9	16
**MGFA* clinical classification**	3	3	2	2
**MG-Composite**	10	13	9	5
**Muscle group for VAS score**	neck	bulbar	right arm	arms
**Medication**	pyridostigmine 120mg/d in 2 doses	prednisone 10mg/d; AZT 150mg/d in 3 doses	pyridostigmine 300mg/d in 5 doses	pyridostigmine 300mg/d in 5 doses
**Thymectomy**	no	yes	no	yes

*MGFA score 0: complete remission of disease
MGFA score 1: pure ocular MG (ptosis and/or diplopia)
MGFA score 2: mild generalized MG
MGFA score 3: moderate generalized MG
MGFA score 4: severe generalized MG
MGFA score 5: respiratory support is needed

3. Therapeutic value

3.1 Beneficial effects

3.1.1 Outcome measures

All primary and secondary outcome measures are presented in Table 2 (page 16).

The QMG has often been used in clinical trials and was recommended as outcome measure for all prospective research in MG by the Myasthenia Gravis Foundation of America (MGFA).[33] The QMG score is a severity score for muscle strength and fatigability consisting of 13 items, each scored from 0 (normal) to 3 (severe weakness). Secondary outcomes include the MG-Composite and the MG-ADL to reflect recent recommendations by the MGFA, as well as a VAS score for subjective assessment of muscle strength, in a muscle group predefined by the patient.[26;34;38] The effect of blinding was assessed during the hospital visit at the end of each cycle by recording which treatment the patient and physician believed was administered during that period.

3.1.2 Motivation for excluding outcome measures

Not applicable

3.1.3 Results of the primary and secondary outcomes of the study

Treatment effect

Add-on treatment with ephedrine compared to placebo resulted in a mean improvement in QMG score of 1.0 (95% confidence interval (CI) 0.21-1.79, Table 2), which was significant for both the trial average treatment effect (p=0.016) and the population treatment effect (p=0.024). Ephedrine treatment also showed a significant trial average treatment effect on all secondary outcome parameters; improving MG-Composite score by 2.7 (p=0.012; 95% CI 0.68-4.65), MG-ADL by 1.0 (p=0.019; 95% CI 0.19-1.81) and a VAS score for individual muscle strength by 1.1 (p=0.033; 95% CI 0.10-2.07). Population average treatment effects for secondary outcomes did not differ significantly from placebo.

Individual treatment effects showed an individual response on QMG and MG-Composite scores for 1 of 4 patients and a significant improvement of MG-ADL and VAS score in another(figure 2 for treatment effect, figure S3 for mean individual scores). Aside from the missing cycle, compliance was 100%, with only small deviations from the planned time schedule. One VAS score was missing, constituting the only missing data point.

Treatment preference of patients within each cycle favoured ephedrine in 6/12 cycles, placebo in 1 cycle and was neutral in 5 cycles. Based on their individual results, three of four patients opted to continue ephedrine treatment in the open label extension phase of the study. The fourth patient discontinued the trial after the randomized cross-over phase because of multiple, individually mild side effects which outweighed the small perceived treatment effect. Although no patient was officially deblinded during the crossover phase, patients correctly guessed presumed treatment arms in 64% of weeks, the investigators correctly guessed 72% of treatment arms. More relevant, in two of the

four N-of-1 trials both patient and investigator correctly guessed every treatment arm.

Discussion and conclusion on beneficial effects:
Previous clinical trials in myasthenia gravis and subsequent analysis have reported a decrease of about 2.6-3.5 points to be clinically significant.[36;39-41] Our current study shows an effect well below the previous cut-off points for a clinically relevant effect. However, no previous studies have tested the effect of a second symptomatic treatment, for which the effect can realistically be expected to be lower than for immunosuppressive drugs.
It can be difficult to extrapolate findings in study group of 4 patients to all similar MG patients. For this reason, we extended our statistical analysis to include a population average treatment effect as well as a trial average treatment effect. The consistent improvement on QMG score for both models, as well as for the trial average treatment effect on secondary outcomes suggests the current findings to be robust. A crossover design could also be confounded by deblinding, or a carry-over effect because of exposure to multiple treatment arms. Although presence of a carry-over effect was not formally tested, QMG and MG-Composite scores were actually slightly worse in placebo periods preceded by ephedrine, as compared to placebo preceded by placebo, making a relevant carry-over effect unlikely. Subjective deblinding by either treatment effect or side effects did occur in the majority of treatment cycles, as recorded by prediction of treatment arms after completed treatment periods during the trial. Correct predictions were however mainly present in the two patients with the smallest effect, also limiting the potential for confounding.

In conclusion, the directly beneficial effects of add-on ephedrine are modest but robust.

Table 2. Treatment effect of add-on ephedrine compared to add-on placebo

	Baseline scores	*Placebo*	*Ephedrine*	*Treatment effect (± 95% CI)*	*P value[a]*	*Popula-tion signifi-cance[b]*
Primary outcome						
QMG (0-39)	10,0 ±4,1	9,5 ± 4,8	8,5 ± 4,5	1,0 (0.21-1.79)	p=0,016	p=0,024
Secondary outcomes						
MG-C (0-50)	9,3 ±3,3	9,1 ±3,6	6,4 ± 5,3	2,7 (0,68-4,65)	p=0,012	p=0,149
MG-ADL (0-24)	3,5 ±0,6	2,8 ± 1,2	1,8 ± 0,8	1,0 (0,19-1,81)	p=0,019	p=0,238
VAS (0-10)	3,8 ±3,1	4,3 ± 2,4	3,2 ± 2,3	1,1 (0,10-2,07)	p=0,033	p=0,198

Mean primary and secondary outcomes expressed as mean ± standard deviation, treatment effect of ephedrine compared to placebo as mean ± 95% confidence interval for trial average treatment effect.
[a] Trial average treatment effect: fixed model assuming normality, no carry-over effects and equal variances for all patients.
[b] Population average treatment effect: mixed model, which additionally assumes a random treatment effect.

3.2 Adverse effects

As can be expected from the mechanism of action of ephedrine, previous studies have reported a dose-dependent increase of blood pressure and / or pulse.[42;43] Ephedrine is also reported to prolong the QT-interval.[44] Therefore, we performed safety measurements during an inpatient visit to study these cardiovascular adverse effects at the current low dose.

Potential adverse effects of ephedrine were planned to be monitored as follows: vital signs to be measured 30, 60 and 120 minutes after administration of study medication on the first day of treatment periods 1 and 2. In addition, an ECG to be recorded 60 minutes after medication is administered on these days, and also during the hospital visit in weeks 1, 2 and 6. Screening blood tests (haematology, liver and renal function tests) would be performed during hospital visits in weeks 1, 2 and 6. Finally, a self-report symptom questionnaire would measure adverse effects on the first day of periods 1 and 2, as well as on the second and fourth day of every period.

Adverse events were limited to mild, transient symptoms (Table 3). Most adverse events were only present during a minority of treatment days. Recurring adverse events were all expected side effects and consisted of palpitations, tremor and restlessness. None of the adverse events required escape medication or extra consultation.

Treatment at the current dose did not show a relevant change in blood pressure or heart rate (see online supplement figure S2A-B), in patients who were all at low risk for cardiovascular disease.

No abnormal ECGS were recorded. Although all conduction intervals, before as well as after treatment, stayed within the normal range, ephedrine significantly prolonged corrected QT (QTc) intervals both at estimated maximum serum concentration on day 1 of treatment, as well as by the end of treatment periods (see appendix figure S4 for QTc intervals).

All four patients completed the trial. One protocol amendment was submitted to and approved by the institutional review board in February 2015, to allow one patient to replace an incomplete cycle.

Discussion and conclusion adverse effects:

There were no dropouts from the trial and adverse events were individually mild and transient. Most adverse events were only present during a minority of treatment days. Recurring adverse events were all expected side effects and consisted of palpitations, tremor and restlessness. None of the adverse events required escape medication or extra consultation.

The literature describes that treatment with ephedrine is not without risk and is associated with cardiovascular events, as well as psychiatric, autonomic and gastrointestinal symptoms, although more severe events have mostly been described at higher doses or in combination with other stimulating drugs.[45;46] In patients with contra-indications, including cardiac disease and risk factors, risks of ephedrine treatment are unlikely to outweigh the small benefit.

Table 3. Adverse events reported in the study. All reported adverse events in the study were mild and only intermittently present.

Adverse events	Ephedrine	Placebo
**Nervous system**		
Tremor hands	2	0
Nervous / restlessness	2	1
Dizziness	1	1
Insomnia	2	1
Muscle cramps	1	0
Headache	1	0
Micturition difficulties	1	0
**Lab abnormalities**		
Leukopenia	1 (3.4)	1 (3.7)
Leukocytosis	1 (11.1)	0
Anemia	1 (7.4)	0
Bilirubinemia	1 (20)	1 (20)
**Cardiovascular**		
Palpitations / tachycardia	3	1
Bradycardia	0	1
**Gastro-intestinal / other**		
Abdominal pain	1	0
Nausea	1	0
Flu-like symptoms	2	1
**Serious adverse events**	0	0
**Adverse events requiring consultation or escape medication**	0	0

3.3 Experience

Ephedrine is not registered for myasthenia gravis anywhere in the world. According to the criteria for judging therapeutic value, experience with an unlicensed medicine is automatically categorized as limited.[22]

On the other hand, oral ephedrine has been prescribed off-label for myasthenia gravis in a limited number of patients since the first case report in 1930, as summarized in our Cochrane systematic review.[8] Ephedrine solution for injection is registered in the Netherlands and is prescribed regularly perioperatively for hypotension. Unfortunately, no exact data are available regarding patient-years of exposure, for either indication.

Currently an open-label phase of the trial described above is ongoing in the LUMC. This will result in pilot data for the long term effects of ephedrine, although the duration and number of included patients is limited.

3.4 Applicability

We list here information from the SMPC of ephedrine tablets registered in Spain (Appendix A1)::

COUNTER INDICATIONS
Coronary thrombosis, high blood pressure, thyrotoxicosis. Patients who are allergic to ephedrine.
PRECAUTIONS
It must be administered with great precaution in: Chronic organic cardiac illnesses, cardiac decomposition or angina pectoris and prostatic hypertrophy.
INCOMPATIBILITIES
M.A.O inhibitors, including during the two weeks after finishing treatment with these drugs.
Digitalis glycosides.
INTERACTIONS
Clorbutol, iodine, silver salts and tannic acids.
WARNING
We warn sportsmen and women that this medicine contains a component which can establish a positive result in an analytic drug test.
SECONDARY EFFECTS
Nervous disorders like :anxiety, insomnia, headaches and dizziness.
Muscular disorders lie: trembling and weakness.
Cardiovascular disturbances such as: tachycardia, palpitations, precordial pain and paleness, especially in hypersensitive patients like neurotics and with hyperthyroidism.
INTOXICATION AND ITS TREATMENT
See SMPC.

Moreover, we note for the sake of completeness that the 1B text of ephedrine HCl for *injection*, which is available in the Netherlands, has additional warnings and interactions (http://db.cbg-meb.nl/IB-teksten/h51937.pdf), e.g. angle-closure glaucoma is a contra-indication and patients with diabetes mellitus should be treated cautiously.

Conclusion on applicability in myasthenia gravis:

A limited subset of patients with myasthenia gravis are expected to benefit from add-on

treatment. Patients with mild disease are likely to respond sufficiently to symptomatic treatment with pyridostigmine. In case of severe disease, more agressive treatment with high-dose immunosuppressants, intravenous immunoglobulin and / or plasmapheresis are indicated, despite possible side effects. In our opinion, ephedrine is only indicated in patients with moderate disease severity, not responding adequately to pyridostigmine and / or low-dose prednisone. All contra-indications are likely to outweigh the small benefit of ephedrine and should in general be regarded as absolute contra-indications.

3.5 Ease of use

Both clinical experience and current evidence support a stable dose of maximally 25mg twice daily.
In normal clinical care, small split tablets can be used, thus assuring the ease of swallowing even for patients with myasthenia gravis.

4. Conclusion and claim

Conclusion and claim

Ephedrine as add-on treatment for myasthenia gravis resulted in a small but consistent improvement of symptoms and weakness. The improvement was consistently found for all primary and secondary outcome parameters. The primary outcome measure, QMG, showed a statistically significant improvement in the trial population and at the population level. The effect size on the primary outcome of the QMG score is small but is likely to be clinically relevant because it concerns an add-on therapy which may postpone or prevent the use of add-on therapies with considerable risks. Although experience with ephedrine for this indication is limited, adverse effects appear to be limited and the applicability of the treatment has been defined to the subset of MG patients with intermediate disease severity and without contraindications. Taken together, an aggregated N-of-1 trial showed that ephedrine is an effective and safe add-on therapy for the subset of myasthenia gravis patients who do not respond adequately to first line treatment but do not yet require high dose immunosuppressive or immunomodulatory therapy.

References

(1) Wockhardt. SPC Ephedrine Hydrochloride tablets. 2008. Accessed 21-7-2015. http://www.medicines.org.uk/emc/medicine/21326
Ref Type: Internet Communication

(2) Gallagher JP, Shinnick-Gallagher P. Ephedrine and neuromuscular transmission, in vivo. Neuropharmacology 1979; 18(10):749-754.

(3) Shinnick-Gallagher P, Gallagher JP. Ephedrine: a postsynaptic depressant drug at the mammalian neuromuscular junction. Neuropharmacology 1979; 18(10):755-761.

(4) Milone M, Engel AG. Block of the endplate acetylcholine receptor channel by the sympathomimetic agents ephedrine, pseudoephedrine, and albuterol. Brain Res 1996; 740(1-2):346-352.

(5) Sieb JP, Engel AG. Ephedrine: effects on neuromuscular transmission. Brain Res 1993; 623(1):167-171.

(6) Engel AG. The therapy of congenital myasthenic syndromes. Neurotherapeutics 2007; 4(2):252-257.

(7) Lashley D, Palace J, Jayawant S, Robb S, Beeson D. Ephedrine treatment in congenital myasthenic syndrome due to mutations in DOK7. Neurology 2010; 74(19):1517-1523.

(8) Vrinten C, van der Zwaag AM, Weinreich SS, Scholten RJ, Verschuuren JJ. Ephedrine for myasthenia gravis, neonatal myasthenia and the congenital myasthenic syndromes. Cochrane Database Syst Rev 2014; 12:CD010028.

(9) Edgeworth H. A report of progress on the use of ephedrine in a case of myasthenia gravis. Journal of the American Medical Association 1930; 94(15):1136.

(10) Schara U, Barisic N, Deschauer M et al. Ephedrine therapy in eight patients with congenital myasthenic syndrome due to DOK7 mutations. Neuromuscul Disord 2009; 19(12):828-832.

(11) Bell DG, Jacobs I, Zamecnik J. Effects of caffeine, ephedrine and their combination on time to exhaustion during high-intensity exercise. Eur J Appl Physiol Occup Physiol 1998; 77(5):427-433.

(12) Bell DG, McLellan TM, Sabiston CM. Effect of ingesting caffeine and ephedrine on 10-km run performance. Med Sci Sports Exerc 2002; 34(2):344-349.

(13) Csajka C, Haller CA, Benowitz NL, Verotta D. Mechanistic pharmacokinetic modelling of ephedrine, norephedrine and caffeine in healthy subjects. Br J Clin Pharmacol 2005; 59(3):335-345.

(14) Meriggioli MN, Sanders DB. Autoimmune myasthenia gravis: emerging clinical and biological heterogeneity. Lancet Neurol 2009; 8(5):475-490.

(15) Vansal SS, Feller DR. Direct effects of ephedrine isomers on human beta-adrenergic receptor subtypes. Biochem Pharmacol 1999; 58(5):807-810.

(16) Keesey JC. Clinical evaluation and management of myasthenia gravis. Muscle Nerve 2004; 29(4):484-505.

(17) Carr AS, Cardwell CR, McCarron PO, McConville J. A systematic review of population based epidemiological studies in Myasthenia Gravis. BMC Neurol 2010; 10:46.

(18) Juel VC, Massey JM. Myasthenia gravis. Orphanet J Rare Dis 2007; 2:44.

(19) Meyer A, Levy Y. Geoepidemiology of myasthenia gravis [corrected]. Autoimmun Rev 2010; 9(5):A383-A386.

(20) Boldingh MI, Maniaol AH, Brunborg C et al. Geographical Distribution of Myasthenia Gravis in Northern Europe - Results from a Population-Based Study from Two Countries. Neuroepidemiology 2015; 44(4):221-231.

(21) Wirtz PW, Nijnuis MG, Sotodeh M et al. The epidemiology of myasthenia gravis, Lambert-Eaton myasthenic syndrome and their associated tumours in the northern part of the province of South Holland. J Neurol 2003; 250(6):698-701.

(22) Farmacotherapeutisch Kompas (FK). Vergoeding niet-geregistreerde indicaties, niet-geregistreerde geneesmiddelen en apotheekbereidingen. 2015. Accessed 21-7-2015.
http://www.farmacotherapeutischkompas.nl/voorna/i/inl%20vergoeding%20van%20niet-geregistreerde%20geneesmiddelen%20en%20indicaties.asp?route=bladeren
Ref Type: Internet Communication

(23) Christensen PB, Jensen TS, Tsiropoulos I et al. Mortality and survival in myasthenia gravis: a Danish population based study. J Neurol Neurosurg Psychiatry 1998; 64(1):78-83.

(24) Padua L, Evoli A, Aprile I et al. Health-related quality of life in patients with myasthenia gravis and the relationship between patient-oriented assessment and conventional measurements. Neurol Sci 2001; 22(5):363-369.

(25) Boldingh MI, Dekker L., Maniaol AH et al. An update on health-related quality of life in myasthenia gravis-results from population based cohorts. Health and Quality of Life Outcomes 2015.

(26) Wolfe GI, Herbelin L, Nations SP et al. Myasthenia gravis activities of daily living profile. Neurology 1999; 52(7):1487-1489.

(27) Skeie GO, Apostolski S, Evoli A et al. Guidelines for treatment of autoimmune neuromuscular transmission disorders. Eur J Neurol 2010; 17(7):893-902.

(28) Hashimoto A, Hanada M, Okada S, Aoki N. A case report of myasthenia gravis associated with Hashimoto's thyroiditis. Folia Psychiatr Neurol Jpn 1981; 35(4):521-525.

(29) McAlpine JK, Thomson JE. Myasthenia gravis and Schmidt syndrome. Postgrad Med J 1988; 64(756):787-788.

(30) Macdonald AM, Keen RI, Pugh ND. Myasthenia gravis and atracurium. A case report. Br J Anaesth 1984; 56(6):651-654.

(31) Vrinten C, Lipka AF, van Zwet EW et al. Ephedrine as add-on therapy for patients with myasthenia gravis: protocol for a series of randomised, placebo-controlled n-of-1 trials. BMJ Open 2015; 5(7):e007863.

(32) Tindall RS, Rollins JA, Phillips JT et al. Preliminary results of a double-blind, randomized, placebo-controlled trial of cyclosporine in myasthenia gravis. N Engl J Med 1987; 316(12):719-724.

(33) Jaretzki A, III, Barohn RJ, Ernstoff RM et al. Myasthenia gravis: recommendations for clinical research standards. Task Force of the Medical Scientific Advisory Board of the Myasthenia Gravis Foundation of America. Neurology 2000; 55(1):16-23.

(34) Burns TM, Conaway M, Sanders DB. The MG Composite: A valid and reliable outcome measure for myasthenia gravis. Neurology 2010; 74(18):1434-1440.

(35) Howard JF, Jr., Barohn RJ, Cutter GR et al. A randomized, double-blind, placebo-controlled phase II study of eculizumab in patients with refractory generalized myasthenia gravis. Muscle Nerve 2013; 48(1):76-84.

(36) Wolfe GI, Barohn RJ, Foster BM et al. Randomized, controlled trial of intravenous immunoglobulin in myasthenia gravis. Muscle Nerve 2002; 26(4):549-552.

(37) Marcus R., Peritz E., Gabriel K.R. On closed testing procedures with special reference to ordered analysis of variance. Biometrika 1975.

(38) Benatar M, Sanders DB, Burns TM et al. Recommendations for myasthenia gravis clinical trials. Muscle Nerve 2012; 45(6):909-917.

(39) Tindall RS, Phillips JT, Rollins JA, Wells L, Hall K. A clinical therapeutic trial of cyclosporine in myasthenia gravis. Ann N Y Acad Sci 1993; 681:539-551.

(40) Katzberg HD, Barnett C, Merkies IS, Bril V. Minimal clinically important difference in myasthenia gravis: outcomes from a randomized trial. Muscle Nerve 2014; 49(5):661-665.

(41) Barohn RJ, McIntire D, Herbelin L et al. Reliability testing of the quantitative myasthenia gravis score. Ann N Y Acad Sci 1998; 841:769-772.

(42) Persky AM, Berry NS, Pollack GM, Brouwer KL. Modelling the cardiovascular effects of ephedrine. Br J Clin Pharmacol 2004; 57(5):552-562.

(43) Haller CA, Jacob P, III, Benowitz NL. Enhanced stimulant and metabolic effects of combined ephedrine and caffeine. Clin Pharmacol Ther 2004; 75(4):259-273.

(44) Milic M, Bao X, Rizos D, Liu F, Ziegler MG. Literature review and pilot studies of the effect of QT correction formulas on reported beta2-agonist-induced QTc prolongation. Clin Ther 2006; 28(4):582-590.

(45) Shekelle PG, Hardy ML, Morton SC et al. Efficacy and safety of ephedra and ephedrine for weight loss and athletic performance: a meta-analysis. JAMA 2003; 289(12):1537-1545.

(46) Haller CA, Benowitz NL. Adverse cardiovascular and central nervous system events associated with dietary supplements containing ephedra alkaloids. N Engl J Med 2000; 343(25):1833-1838.

EFEDRINA LEVEL
Ephedrine hydrochloride

COMPOSITION
Per tablet:
Ephedrine hydrochloride.............50mg
Excipients: microcrystalline cellulose, talc, magnesium stearate and carboxymethyl starch.

INDICATIONS
Bronchial asthma, hay fever (coryza hays).
Contributes to treatment of: migraines, urticarias, eczema, neurodermititis, Quinke's edema etc. Bronchial spasms in acute bronchial asthma, spastic bronchitis, lung emphysema, asthma states and severe chronic asthma.

DOSAGE
The usual dose except in the case of facultative prescriptions is one tablet 2-3 times a day.

COUNTER INDICATIONS
Coronary thrombosis, high blood pressure, thyrotoxicosis. Patients who are allergic to ephedrine.

PRECAUTIONS
It must be administered with great precaution in: Chronic organic cardiac illnesses, cardiac decomposition or angina pectoris and prostatic hypertrophy.

INCOMPATIBILITIES
M.A.O inhibitors, including during the two weeks after finishing treatment with these drugs.
Digitalis glycosides.

INTERACTIONS
Clorbutol, iodine, silver salts and tannic acids.

WARNING
We warn sportsmen and women that this medicine contains a component which can establish a positive result in an analytic drug test.

SECONDARY EFFECTS
Nervous disorders like :anxiety, insomnia, headaches and dizziness.
Muscular disorders lie: trembling and weakness.
Cardiovascular disturbances such as: tachycardia, palpitations, precordial pain and paleness, especially in hypersensitive patients like neurotics and with hyperthyroidism.

INTOXICATION AND ITS TREATMENT
Insomnia and other stimulating effects of ephedrine on the central nervous system can be counteracted by the administration of hypnotics.
In case of severe intoxication the stomach must immediately be cleaned by aspiration and flushing. To control excitement levels sedatives like chlopromacine can be

administered with a dose of 50 or 100mg intramuscularly or as a barbiturate. To control tachycardia propanole can be administered intravenously with a dose of 2.5-5mg, or with practolole in asthmatic patients. The elimination of ephedrine must be helped by acidifying the urine with sodium chloride.
In the case of overdose or accidental ingestion consult the Toxicolgy Information Service. Telephone: 915620420.

PRESENTATION
Box with 24 tablets

WITH MEDICAL PRESCRIPTION
MEDICINES MUST BE KEPT OUT OF REACH OF CHILDREN

Holder of the authorisation for the commercialisation and fabrication of the product.

LABORATORIOS ERN, S.A. Pedro IV, 499 - 08020 Barcelona, España
Tel: 93 314 80 11   Fax: 93 314 40 96

Responsable for fabrication.
Laboratorios ERN, S.A. Gorcs i LLadó, 188 – 08210 Barberá del Vallés, Barcelona. España.
Laboratorios ERN, S.A. Perú, 228 – 08020 Barcelona. España.

MANUFACTURER'S AUTHORISATION [1, 2]

1. Authorisation Number	0152
2. Name of authorisation holder	LABORATORIOS ERN, S.A.
3. Address(es) of manufacturing site(s)	LABORATORIOS ERN, S.A., C/ Peru 228, Barcelona, Barcelona, 08020, Spain<br>LABORATORIOS ERN. S.A., C/ Gorcs i Llado, 188, Barberà del Valles, Barcelona, 08210, Spain
4. Legally registered address of authorisation holder	C/ Pedro IV, 499, Barcelona, Barcelona, 08020, Spain
5. Scope of authorisation and dosage forms [2]	ANNEX 1 and/ or ANNEX 2
6. Legal Basis of authorisation	Art. 40 of Directive 2001/83/EC
7. Name of responsible officer of the competent authority of the member state granting the manufacturing authorisation	confidential
8. Signature	
9. Date	2012-04-23
10. Annexes attached	Annex 1 and/or Annex 2<br>Optional Annexes as required:<br>Annex 3 (Addresses of Contract Manufacturing Site(s))<br>Annex 4 (Addresses of Contract laboratories)<br>Annex 5 (Name of Qualified Person)<br>Annex 6 (Name of responsible persons)<br>Annex 7 (Date of inspection on which authorisation granted, scope of last inspection)<br>Annex 8 (Manufactured/ imported products authorised) [3]

[1] *The authorisation referred to in paragraph 40(1) of Directive 2001/83/EC and 44(1) of Directive 2001/82/EC, as amended, shall also be required for imports coming from third countries into a Member State.*

[2] *Guidance on the interpretation of this template can be found in the Help menu of EudraGMDP database.*

[3] *The Competent Authority is responsible for appropriate linking of the authorisation with the manufacturer's application (Art. 42(3) of Directive 2001/83/EC and Art. 46(3) of Directive 2001/82/EC as amended).*

EudraGMP
EudraGMP

SCOPE OF AUTHORISATION — ANNEX 1

Name and address of the site : LABORATORIOS ERN, S.A., C/ Peru 228, Barcelona, Barcelona, 08020, Spain

Human Medicinal Products

**Authorised Operations**
MANUFACTURING OPERATIONS (according to part 1)

**Part 1 - MANUFACTURING OPERATIONS**	
**1.2**	**Non-sterile products**
	1.2.1 Non-sterile products (processing operations for the following dosage forms)<br>1.2.1.5 Liquids for external use<br>Special Requirements<br>9 Other: None(en)<br>1.2.1.6 Liquids for internal use<br>Special Requirements<br>9 Other: None(en)<br>1.2.1.13 Tablets<br>Special Requirements<br>9 Other: None(en)

EudraGMP

SCOPE OF AUTHORISATION **ANNEX 1**

Name and address of the site : LABORATORIOS ERN. S.A., C/ Gorcs i Llado, 188, Barberà del Valles, Barcelona, 08210, Spain

Human Medicinal Products

**Authorised Operations**
MANUFACTURING OPERATIONS (according to part 1)

Part 1 - MANUFACTURING OPERATIONS

**1.2**	**Non-sterile products**
	1.2.1 Non-sterile products (processing operations for the following dosage forms)<br>1.2.1.1 Capsules, hard shell<br>Special Requirements<br>9 Other: None(en)<br>1.2.1.8 Other solid dosage forms: other.solid.dosage.forms(en)<br>Special Requirements<br>9 Other: None(en)<br>1.2.1.11 Semi-solids<br>Special Requirements<br>9 Other: None(en)<br>1.2.1.13 Tablets<br>Special Requirements<br>9 Other: None(en)

EudraGMP

Supplemental figures.

Figure S1 Flow Diagram of recruitment, follow-up, and analysis

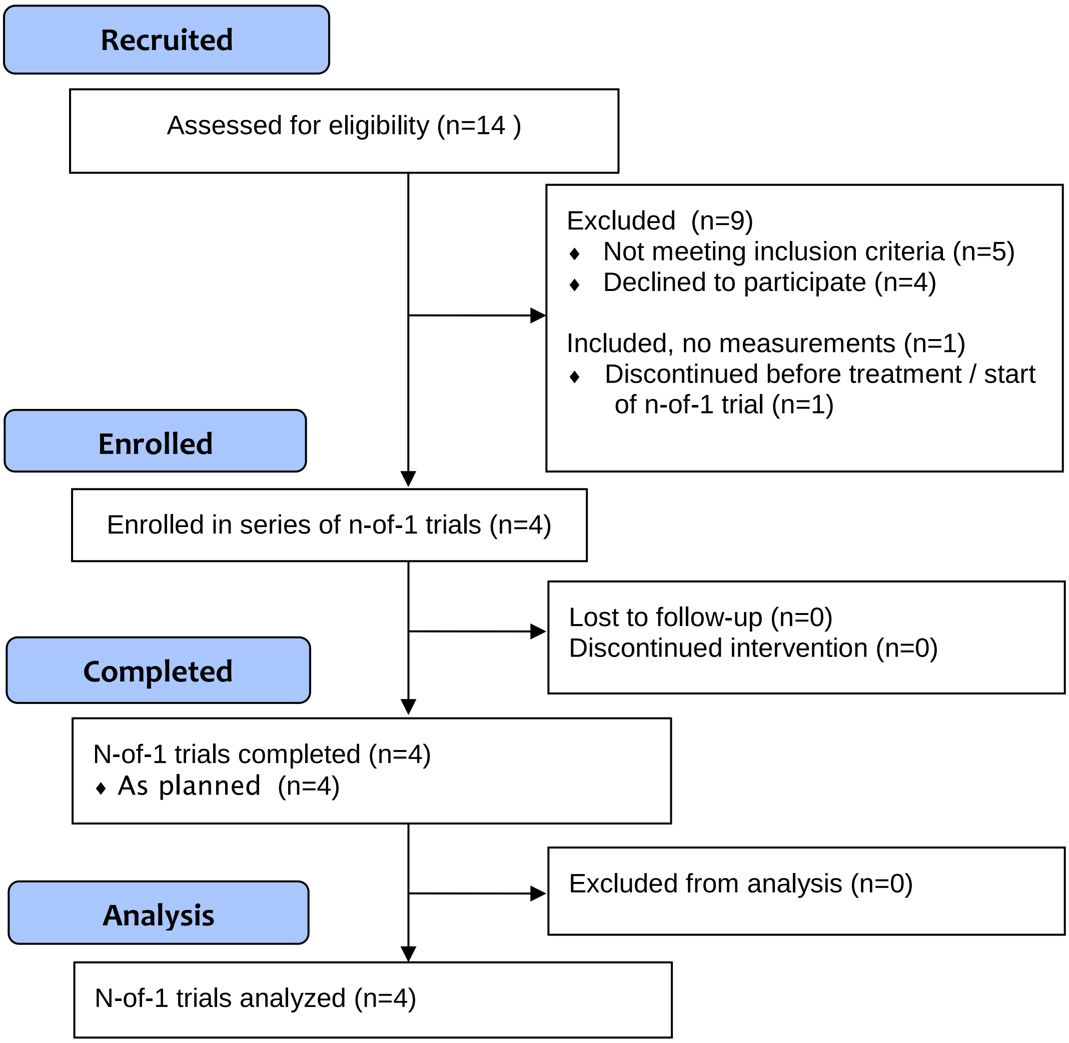

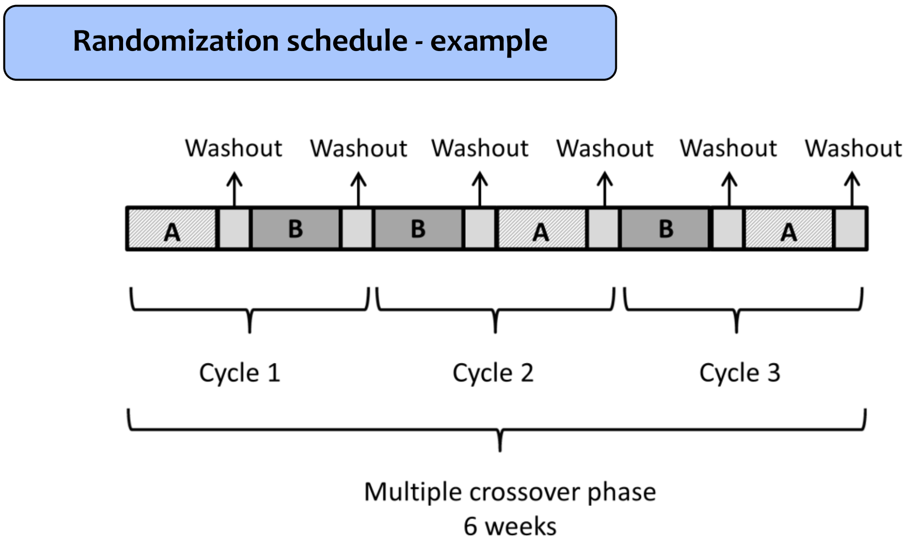

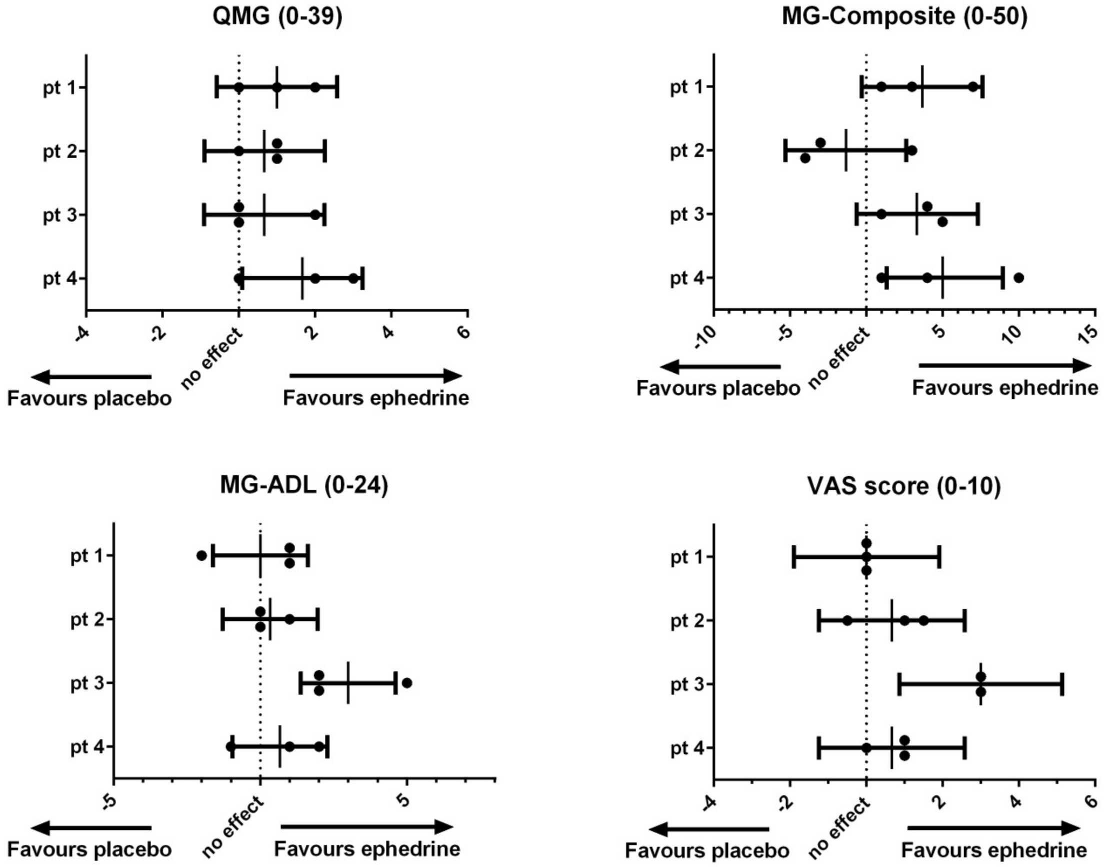

Figure S2. Mean treatment effect per cycle for each patient. Each patient has completed 3 treatment cycles, for which the benefit of ephedrine treatment compared to placebo is shown. Error bars represent 95% confidence interval, which are equal for all patients due to the assumption of equal variances in the statistical model.

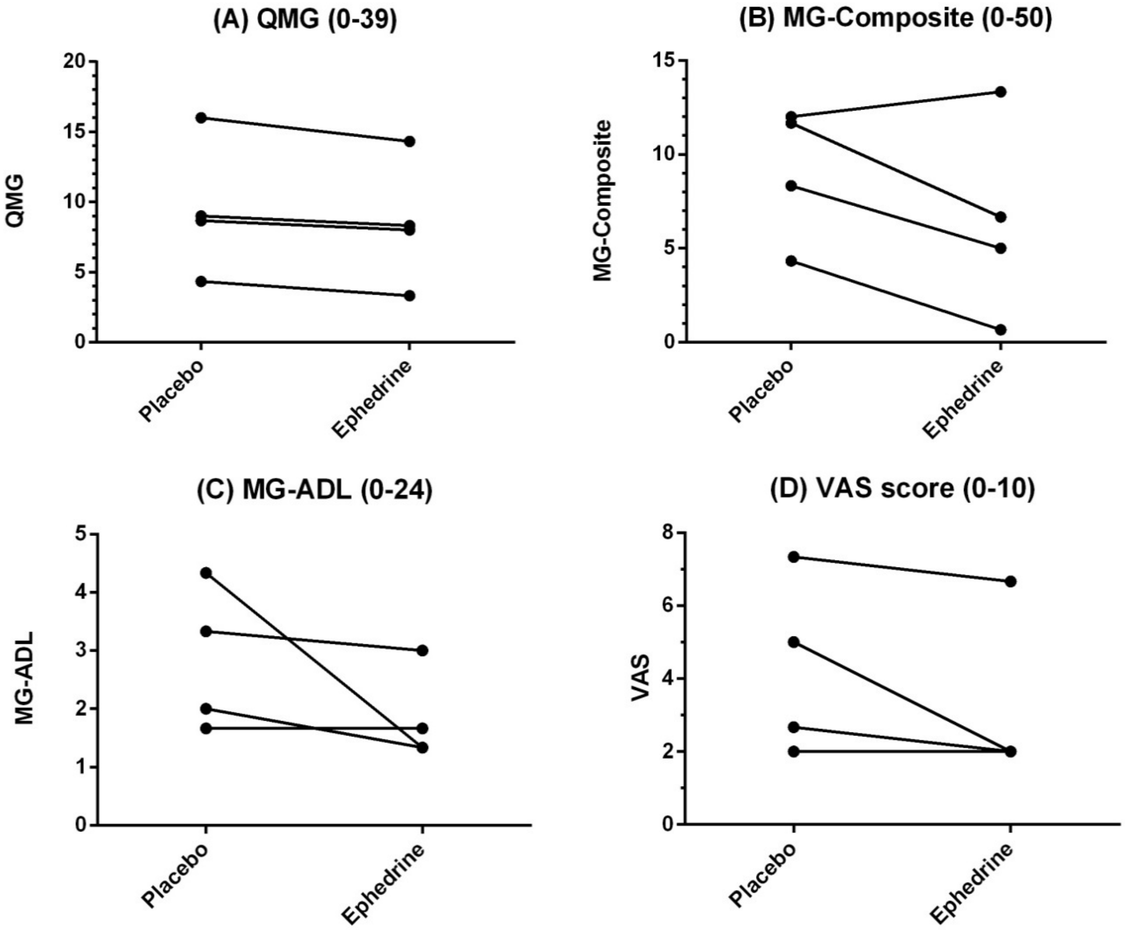

Figure S3. Individual mean scores during ephedrine and placebo treatment.
A Individual mean QMG scores; B MG-Composite scores, C MG-ADL scores, D VAS scores of muscle group chosen by the patient.

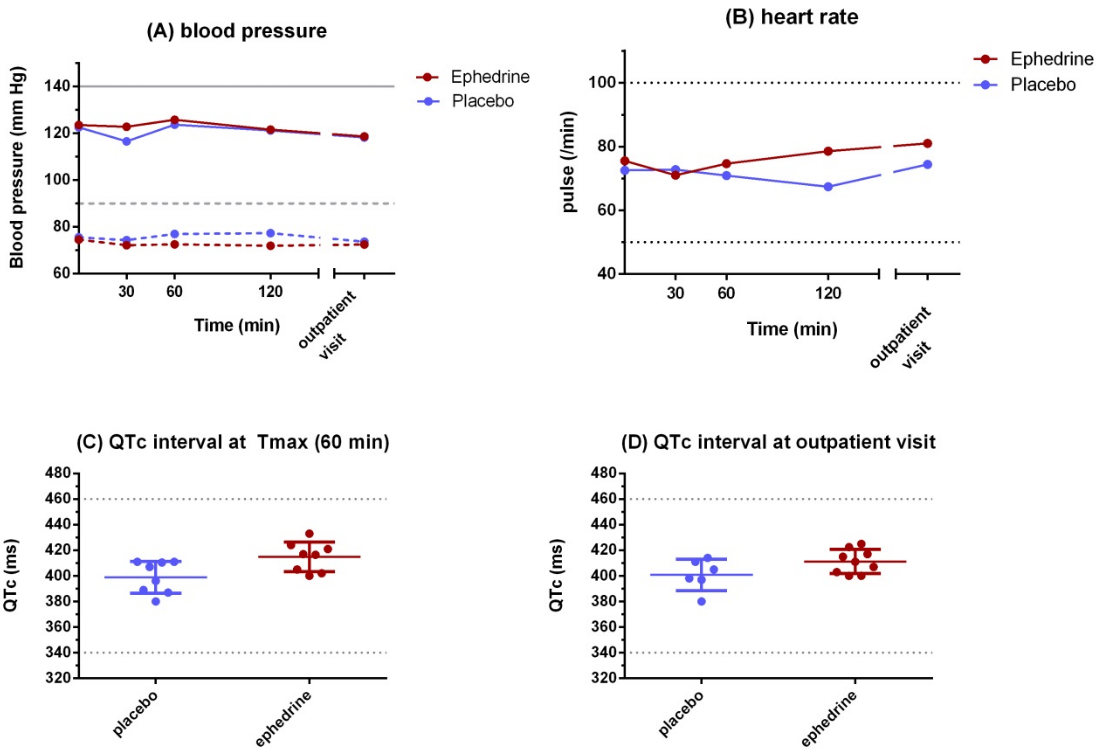

Figure S4. Cardiovascular effects of ephedrine compared to placebo.

(A) Systolic (solid lines) and diastolic (dashed lines) blood pressure during ephedrine (red lines) and placebo (blue lines) treatment.
Mean blood pressure at outpatient visit (day 5 of treatment period) is also shown.

(B) Heart rate at baseline, 30, 60 and 120 minutes after start of ephedrine or placebo treatment. These were measured at day 1 of week 1 and 2 for all patients.
Mean heart rate at outpatient visit (day 5) is also shown.

(C) Corrected QT interval 60 minutes (estimated time of maximum plasma concentration) after start of ephedrine or placebo treatment, measured at day 1 of week 1 and 2.

(D) Corrected QT interval at outpatient visit (day 5 of treatment period).

agencia española de medicamentos y productos sanitarios

Presentación Acceso a la aplicación Búsqueda por descripción clínica Nomenclátor de prescripción Registro de medicamentos Glosario

Inicio - Medicamentos de uso humano

› Centro de Información online de Medicamentos de la AEMPS - CIMA

Información del Medicamento:

Número de Registro:	15544	Medicamento:	EFEDRINA COMPRIMIDOS
Laboratorio titular:	LABORATORIOS ERN, S.A.	Estado del medicamento:	Autorizado 01/05/1933
Principios Activos:	EFEDRINA HIDROCLORURO		
Clasificación ATC:	Nivel 3: R03C - ADRENÉRGICOS PARA USO SISTÉMICO		
	Nivel 4: R03CA - Agonistas de receptores adrenérgicos alfa y beta		
	Nivel 5: R03CA02 - Efedrina		

Listado de Formatos:

Código Nacional	Nombre del Formato	Estado del formato del Medicamento	Comercialización	Comentarios
749622	EFEDRINA COMPRIMIDOS, 24 comprimidos	Autorizado 01/05/1933	Comercializado	

Exportar resultados a: CSV Excel XML

Volver

Para reportar incidencias informáticas relativas a CIMA (No se atenderán consultas médicas) envíe un correo a sugerencias_FT@aemps.es.

Visite http://www.aemps.gob.es/informa/info-atencion-ciudadano/home.htm para otras direcciones de correo de la AEMPS.

Aplicación para dispositivos móviles de la AEMPS con información de medicamentos de uso humano

WCAG WAI AA CERTIFICACION euroCert W3C XHTML 1.0 W3C CSS W3C WAI-AA WCAG 2.0

© Agencia Española de Medicamentos y Productos Sanitarios
